# Unprecedented Route to Amide-Functionalized Double-Decker Silsesquioxanes Using Carboxylic Acid Derivatives and a Hydrochloride Salt of Aminopropyl-DDSQ

**DOI:** 10.1021/acs.inorgchem.2c04546

**Published:** 2023-03-29

**Authors:** Anna Władyczyn, Łukasz John

**Affiliations:** Faculty of Chemistry, University of Wrocław, 14 F. Joliot-Curie, 50-383 Wrocław, Poland

## Abstract

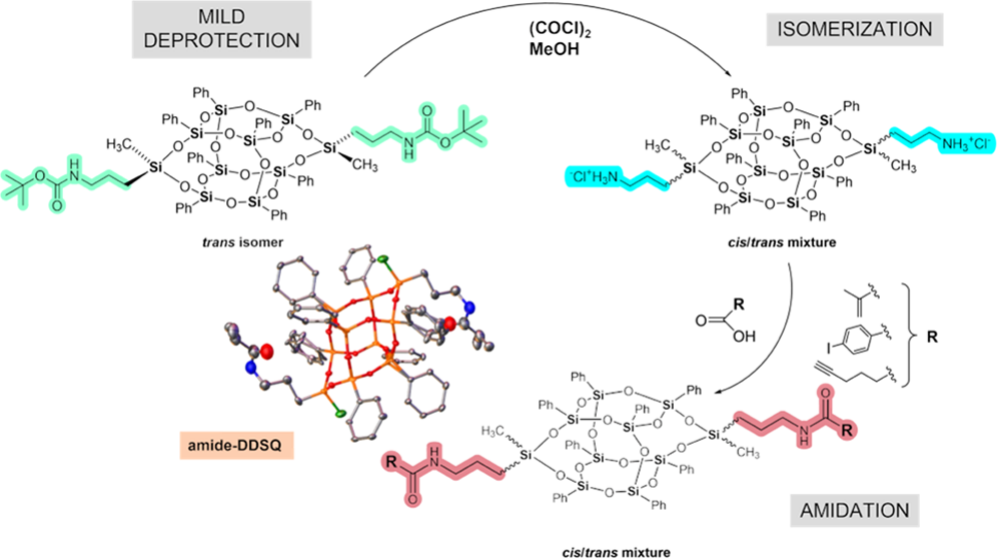

An easy, efficient, and scalable synthetic procedure is described to obtain novel amide-functionalized double-decker silsesquioxanes (DDSQs). The use of mild conditions of deprotection of the BOC group, which does not result to the cleavage of the cage-like silsesquioxane structure, is reported. This method leads to the so far undescribed hydrochloride salt of aminoalkyl-DDSQ. Interestingly, the *cis*/*trans*-isomerization of DDSQ molecules was observed during the reaction. The resulting compounds are characterized using multinuclear NMR (^1^H, ^13^C, and ^29^Si), MALDI-TOF, FT-IR, and elemental analysis. Moreover, crystal structures are reported for three *trans* DDSQs. The chloride salt of aminoalkyl derivative, obtained in one of the steps of the synthetic pathway, shows an intriguing structure of the crystal lattice in which large channels are present, caused by ionic interactions in the lattice. The described approach opens the way to synthesizing new DDSQ derivatives and materials using BOC-blocked amines. We believe our findings would advance investigations about new materials based on little known organic–inorganic DDSQ-based hybrids.

## Abstract 2

This article reports on the approach that opens the way to synthesizing amide-DDSQ derivatives and materials using aminopropyl-DDSQ and carboxylic acids.

## Introduction

Rapid buildout of synthetic and analytical techniques permits chemists to obtain hybrid architectures with mixed properties of organic and inorganic segments. The advanced approach to obtaining new hybrids is based on combining proper molecules with different properties at the molecular level rather than simply yielding a mixture of components. Nowadays, this approach is expected to be an effective way to obtain novel high-performance polymeric materials with precisely designed properties due to structural control at the molecular level.^1−6^ The use of preformed hybrid molecules paves the way to so-called “Lego chemistry”, which allows the creation of custom materials or hybrid assemblies. In this step-by-step procedure, similar to Lego bricks, hybrid molecules come together to form a material allowing better control of its structure on the semilocal scale.^7^

Polyhedral oligomeric silsesquioxanes (POSS) constitute a perfect example of a hybrid architecture that fits into the convention of molecular building blocks for advanced materials.^8^ Among this group of silsesquioxanes, most attention is attracted to T_8_-type structures containing eight silicon atoms connected by oxygen bridges that form a cage-like architecture. These compounds have an inorganic siloxane cage-like core and organic functional side arms that can be modified. Unfortunately, the selective obtaining of such species with multiple functional groups remains challenging because of the formation of, among others, cage-like structures with lower symmetry (e.g., T_10_ and T_12_ cages) or open-like cages.^9−13^

**Figure 1:**
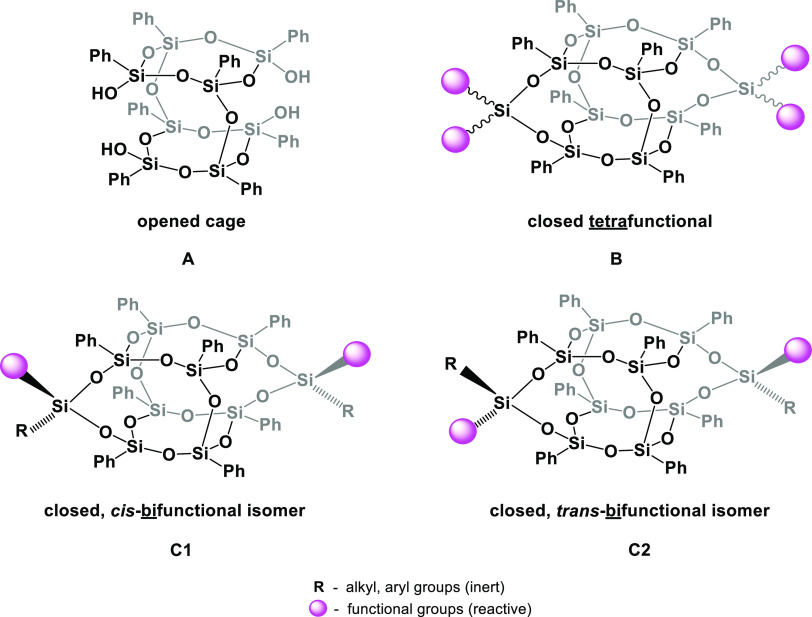
Possible architectures of the closed-type DDSQs: (A) opened cage; (B) closed tetrafunctional; (C1) closed, *cis*-bifunctional; and (C2) closed, *trans*-bifunctional.

In this field of interest, double-decker silsesquioxanes (DDSQs) seem promising hybrid molecules with vast potential for precisely controlled design tailor-made for the “Lego chemistry” approach. DDSQ is composed of two tetracyclosiloxane decks connected via two oxygen bridges. Each silicon atom in the molecule’s core is linked to an inert phenyl group. The side part of the silsesquioxane core consists of lateral silicon atoms with organic chains, most often terminated with functional groups. Depending on the silane used in the silylation reaction, DDSQ adopts two possible architectures—opened and closed (Figure 1).^14,15,36^

A closed, bifunctional DDSQ structure exists in *cis* and *trans* configurational isomers (Figure 1C1, C2). Separation of those geometrical isomers is possible due to the fractional crystallization approach.^36^ Also, in our previous study,^16^ the method of synthesis and effective separation of unique hydroxyalkyl-substituted DDSQs was reported in detail. Furthermore, silsesquioxanes are still very interesting in polymer chemistry because their addition to the polymeric matrix improves the materials’ physical, biological, and chemical properties.^17−21^

Amine-functionalized POSS compounds are desirable substrates due to the versatility of their possible functionalization with many reactants—like carboxylic acids, esters, anhydrides, carbonates, isocyanates, acrylates, and epoxides as well as reactants suitable for nucleophilic substitution.^22^ To date, the synthesis of various POSS derivatives functionalized with an alkylamino group has been developed, e.g., octa(3-aminopropyl)silsesquioxane (H_2_Npropyl)_8_POSS^22−24^ (and also its hydrochloride^25,26^ and trifluoromethanesulfonate salt^27^), (3-aminopropyl)heptaisobutylsilsesqioxane (H_2_Npropyl)heptaisobutylPOSS,^28^ bi(3-aminopropyl)hexaisobutylsilsesqioxane,^29^ and 3,13-bi(3-aminopropyl) DDSQ^34^ (and its BF_3_-complexed derivative^38^).

Synthesis of amides is an already used strategy for functionalizing silsesquioxanes with amino groups. It is an easy, scalable, and efficient modification method that allows to decorate the vertices groups with various substituents. Männle et al.^22^ obtained amido-POSS using (H_2_Npropyl)_8_POSS, hexanoic acid, and 1-propoxy-2-propanol. Feher et al.^39^ used (H_2_Npropyl)_8_POSS and its HCl salt in many transformations, e.g., amidation using benzoyl chloride and *N*,*N*-diisopropylethylamine in DMF; amidation with succinic and maleic anhydrides in dry methanol; and addition of lactones in DMSO. Our group proposed a method^40^ of obtaining amido-functionalized POSS using acyl chlorides in the presence of triethylamine.

Interestingly, eight amino groups at the POSS corners enabled Kozuma et al.^41^ to obtain polyamides by reaction with polyacids in the presence of EDC and NHS using DMSO as a solvent. There are also many examples of utilizing (H_2_Npropyl)heptaisobutylPOSS in amide synthesis, e.g., Wang et al.^42^ described a reaction with acrylic acid in the presence of DCC and DMAP in THF as a solvent; Wang et al.^42^ obtained azobenzene-tethered POSS in a condensation reaction with azobenzene chlorides in the presence of NEt_3_ in dichloromethane. Also intriguing is the approach that uses multifunctional chlorides to create structures where the cages are connected. Hou et al.^43^ presented structures obtained by condensation of bifunctional acid chlorides and (H_2_Npropyl)heptaisobutylPOSS with NEt_3_ as a base and dichloromethane as a solvent. As we showed recently,^44,45^ interesting derivatives can be obtained using trifunctional acid chlorides with the addition of 2-picolylamine and dichloromethane as a solvent.

**Scheme 1:**
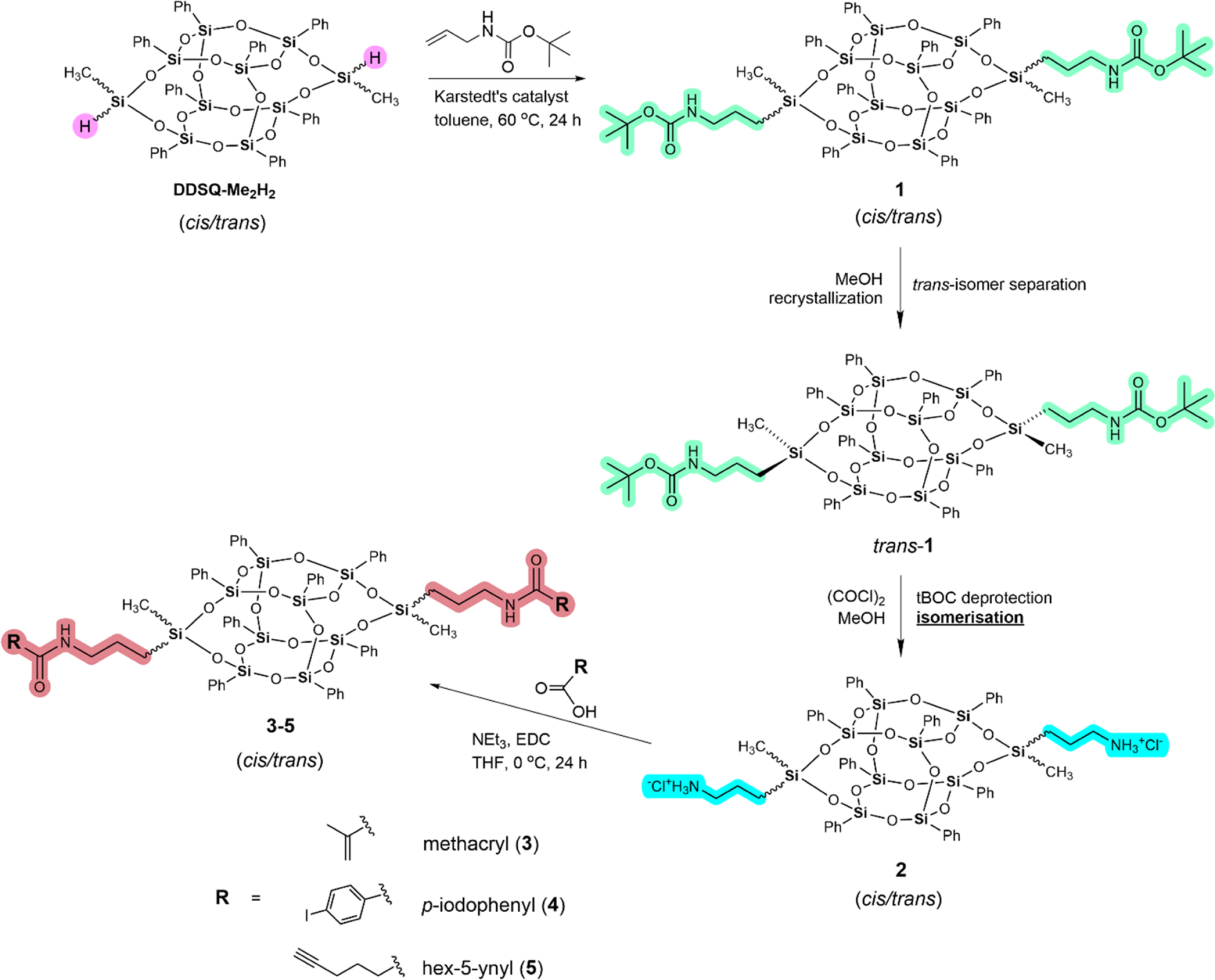
Synthesis of Amide-Functionalized DDSQ **3–5**

Herein, we aimed to obtain aminoalkyl-functionalized DDSQ (DDSQ-(NH_2_)_2_) and use it as a substrate in the amidation reaction. Considering the instability of the silsesquioxane core in the presence of water and nucleophiles,^30−33,44,49−51^ we developed a simple and elegant method of obtaining DDSQ-(NH_2_)_2_ hydrochloride salt (DDSQ-(NH_3_Cl)_2_) by deprotection of the BOC group under mild conditions. According to our best knowledge, this is the first report on synthesizing the hydrochloride salt of bi(3-aminopropyl) DDSQ. Moreover, we propose a practical approach to obtain new amide-functionalized DDSQ using carboxylic acid derivatives and DDSQ-(NH_3_Cl)_2_ as substrates, which could be used as a universal bifunctional linker in polymer synthesis (Scheme 1).

## Results and Discussion

**Figure 2:**
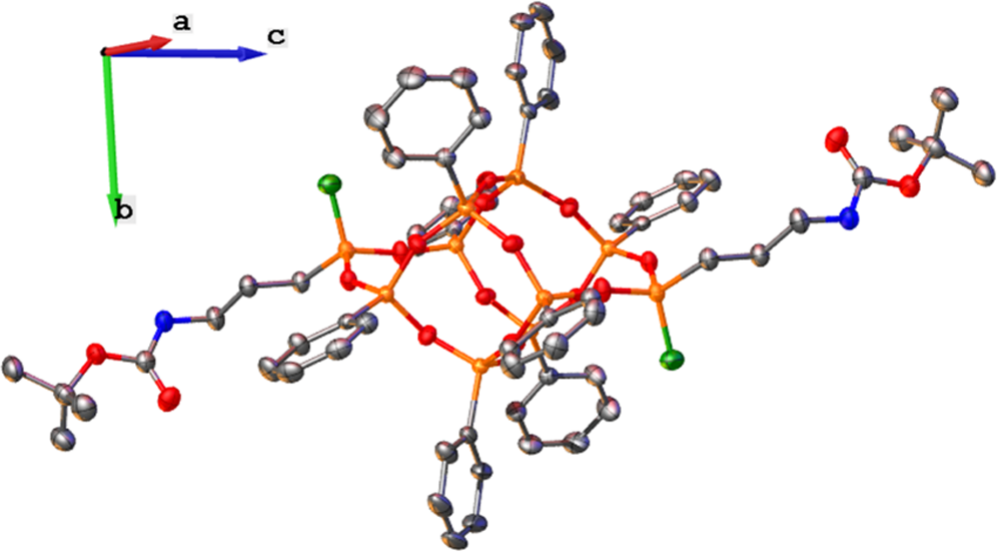
Crystal structure of *trans-***1**: silver, carbon; orange, silicon; red, oxygen; blue, nitrogen; and light green, carbon from the methyl group. Thermal ellipsoids are shown at a 50% probability level. All hydrogen atoms are omitted for clarity.

The aforedescribed 3,13-bi(3-tert-butoxycarbonylaminopropyl) DDSQ **1** was applied as a substrate for obtaining 3,13-bi(3-aminopropyl) hydrochloride DDSQ **2**. Some modifications of the procedure of the synthesis of **1** described by Ishida et al.^34^ were applied; more precisely: using toluene at 60 °C instead of THF at 40 °C, extending the reaction time from 4 to 24 h, using the reduced equivalent of alkene in hydrosilylation reaction (from approximately 4 to 2.5 per DDSQ). These modifications resulted in yield increase, substrates amount reduction, and shortening of the purification step (column chromatography is not required). The conversion of **DDSQ-Me**_**2**_**H**_**2**_ was determined by ^1^H NMR spectroscopy by monitoring the characteristic signal from the Si–H proton at δ = 4.98 ppm.^16^ It is worth noting that the reaction occurred selectively, and any possible side products were not identified in the post-reaction mixture, such as isomerized alkenes, α-adducts, or dehydrogenative silylation products. Moreover, we performed *trans*-**1** separation using a straightforward fractional crystallization method using cold methanol (the corresponding *trans*-**1** was obtained with a 71% isolated yield). As previously observed for other DDSQ derivatives,^16,35,37,41,46^ the *trans*-isomer appears as an excess in the post-reaction mixture; its poorer solubility is probably due to the tighter packing of molecules in the lattice. Recently, we have shown that *cis*-isomers can arrange intermolecular interactions to form dimers, while *trans*-isomers create 1D polymers.^16^ Successful separation of the pure *trans*-isomer was undoubtedly confirmed by NMR (^1^H, ^13^C, and ^29^Si), MALDI-MS, FT-IR, and TG-DTA (see Figures S1–S6). The direct evidence of the siloxanes core geometry is the number of signals in the ^29^Si NMR spectrum—for *trans*-**1** chemical shifts at δ = −17.79 (SiCH_3_), −78.53, and −79.52 (Si–O–Si-Ph) ppm (Figure S3) and for *cis*-**1** at δ = −17.28 (SiCH_3_), −76.92, −78.02, and −78.99 (Si–O–Si-Ph) ppm (Figure S4). All found chemical shifts stay in agreement with the literature.^35^ Moreover, valuable proof of obtaining the *trans* form is their crystal structure (Figure 2 and Table S1). Diffraction data analysis reveals that *trans*-**1** crystallizes in the monoclinic (*P*2_1_/*c*) space group with half of the molecule occupying an asymmetric unit; the value for the C–Si–Si–C pseudo-torsion angle (180°) indicates obtaining the *trans*-isomer. The lengths of the Si–O bonds are consistent with the literature data, with an average value of 1.6 Å.^16,34,36,38,48,52−54^ It is noteworthy that there is no disorder in the structure, and the quality of the solution is high; *R*_1_ and w*R*_2_ coefficients equal 3.49 and 8.91%, respectively. In the DDSQ structures described so far, disorders and relatively high reliability factors are pretty common.^16,34,36,38,48,52^ The only intermolecular interactions in the crystal lattice are π-stackings between phenyl groups of neighboring molecules.

Because it has been proven that DDSQs’ isomers can differ in physicochemical properties, such as solubility and melting temperature,^26^ we decided to use the pure *trans*-**1** in further transformations.

So, in the next step, *trans*-**1** was allowed deprotection of the tert-butoxycarbonyl (N-BOC) group. It is necessary to emphasize that performing deprotection on the N-BOC-functionalized DDSQ is not a trivial procedure because typical conditions of the reactions are harsh. There is a need to use highly concentrated strong acids, such as hydrochloric acid, sulfuric acid, orthophosphoric acid, or trifluoroacetic acid (TFA), and nucleophilic reagents, such as tetra-*n*-butylammonium fluoride (TBAF) and trimethylsilyl iodide (TMSI). Unfortunately, such conditions are well-known factors that can cause rearrangement and/or cleavage of the siloxane core structure.^30−32^ Initially, the method proposed by Ishida *et al.* was tested.^34^ This protocol describes deprotection using TFA. Due to the lack of information regarding TFA’s concentration, we decided to examine different concentrations of trifluoroacetic acid (50, 10, and 1%) and the reaction time (1, 4, 12, and 24 h). However, we could not obtain the product with a satisfactory yield (<50%) in any tested conditions. The reaction proceeded very slowly for low concentrations and short times; for concentrated solutions, cleavage of the Si–O skeleton was observed, manifested in the ^1^H NMR spectrum by an increase in the number of signals and their significant broadening.

After that, we decided to test the mild deprotection using oxalyl chloride in methanol. It turned out that the approach proposed by George et al.,^47^ which we slightly modified, brought the expected results. This procedure provides the complete deprotection of the N-BOC group of *trans-***1** after 24 h without the decomposition of the cage-like core. Interestingly, contrary to the tested compounds described in the abovementioned publication,^47^*trans*-**1** does not dissolve in methanol; it forms a white suspension mixture. Furthermore, we also tested using other solvents in this reaction, in which *trans*-**1** is well soluble, i.e., THF, dichloromethane, chloroform, or acetonitrile. We also checked the reaction results for mixtures of these solvents with methanol. However, it turns out that the best conversion rate was obtained in pure methanol. This brings us to the conclusion that the solubility of the substrate is not crucial for the success of the reaction. It rather seems that the presence of methanol is a pivotal factor—which is consistent with the authors’ observations.^47^

Moreover, we proposed a convenient and straightforward method for the purification and isolation of **2** in a crystalline form; from the post-reaction mixture, concentrated on a rotary evaporator, the pure product can be obtained by recrystallization from water. This methodology is sufficient because **2** is insoluble in water, whereas dimethyl oxalate (byproduct) dissolves in water (Figure S33 and Table S2). Therefore, we opt that the pivotal influence on the success of the reaction is that HCl is generated in situ. Presumably, the concentration of HCl during the reaction is low enough to leave the integrity of the inorganic core and, on the other hand, high enough to deprotect the N-BOC group effectively. Furthermore, during the attempts to optimize the reaction, it was observed that the ratio of oxalyl chloride to the substrate was not as critical as we thought. In our opinion, the key point is the concentration—oxalyl chloride should be added to methanol suspension in an amount in which its concentration will be at least 0.1 M. We noted that when the concentration is lower, only partial deprotection occurs during 24 h. It turns out that DDSQ **2** was obtained in 75% yield as the hydrochloride salt, proving that HCl generates in situ. Because **2** is decorated with polar, ionic groups, its solubility in most organic solvents (e.g., chloroform, hexane, dichloromethane, diethyl ether, acetonitrile) is poor; it exhibits slight solubility only in methanol, DMF, and THF. Considering the instability of amine silsesquioxane, already proven by Feher et al.^23^ and confirmed by our group,^26^ obtaining this derivative as a hydrochloride salt allows longer storage of the compound after synthesis in air conditions.

The product’s structure was confirmed by multinuclear NMR (^1^H, ^13^C, and ^29^Si), FT-IR, MALDI-MS, TG-DTA, and X-ray diffraction analysis (Figures S8–S14 and Table S1). Unexpectedly, ^29^Si NMR suggests that during this step, DDSQ **2** isomerizes, which leads to the mixture of *cis*/*trans* products. This phenomenon has never been reported in the literature. The presence of two isomers in the mixture is indicated by the number of signals in the ^29^Si NMR spectrum (Figure S11) at δ = −18.15 (*cis*/*trans)* (SiCH_3_), −78.12 (*cis*/*trans)*, −78.90 (*cis*), −79.11 (*trans*), and −79.32 (*cis*) (Si–O–Si-Ph) ppm. The doubling of the ^1^H NMR signal at δ = 0.43 ppm of the protons of the methyl group (SiCH_3_) is also symptomatic (Figure S8). The mechanism of such a transition remains unknown and requires further experimental confirmation. We hypothesize that the isomerization can be driven by the chloride anion, which can undergo substitution on the lateral Si atom. An unstable intermediate containing a pentavalent Si atom may be formed if this occurs. In the next step, nucleophile leaving would generate an interchange in the position of the Si–CH_3_ group and the alkyl substituent. The possibility of the existence of such a perturbation for pentavalent Si has already been postulated based on the results of density functional theory calculations by Couzijn et al.^51^

**Figure 3:**
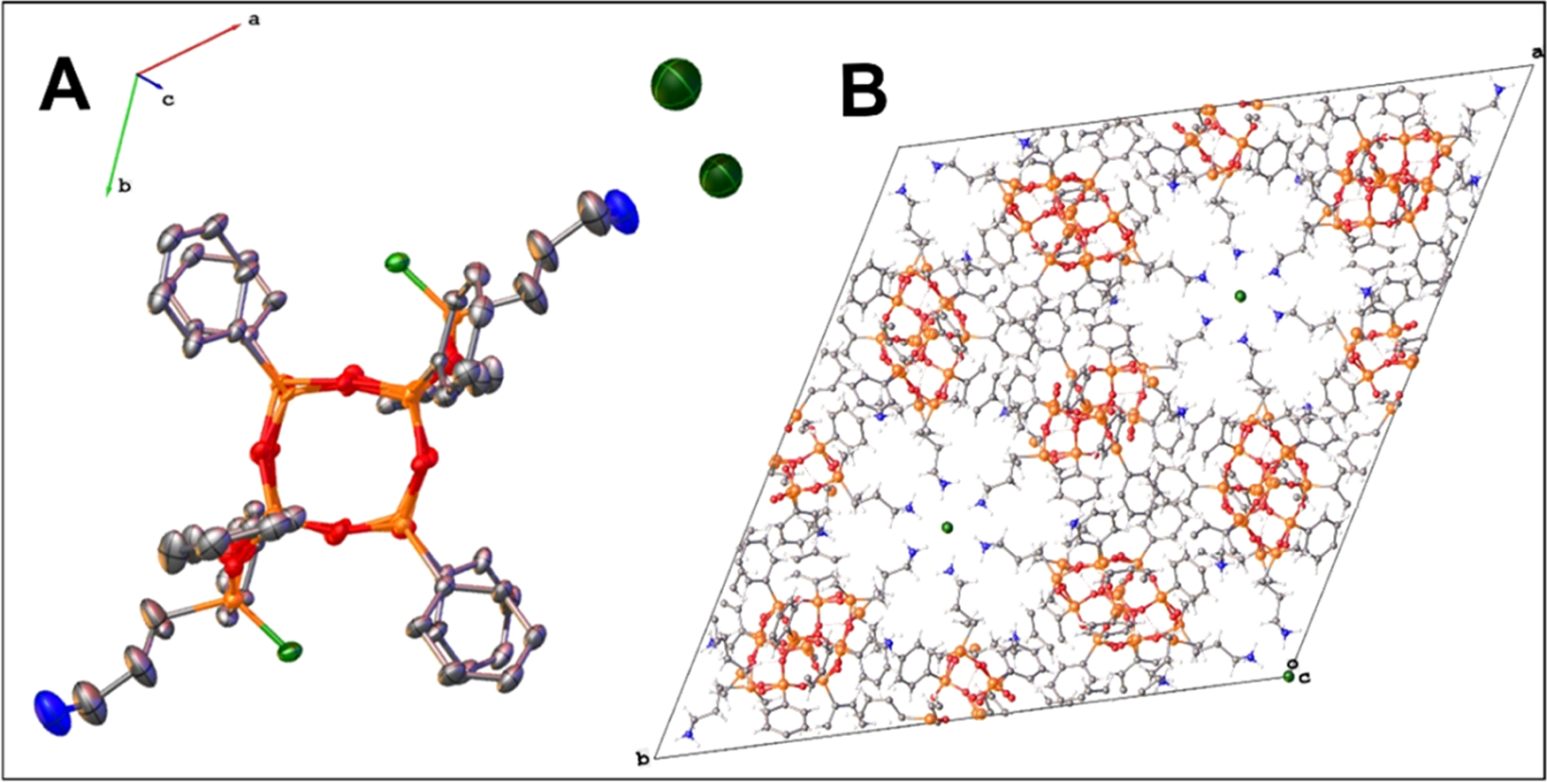
(A) Crystal structure of *trans*-**2**: silver, carbon; orange, silicon; red, oxygen; blue, nitrogen; dark green, chloride; and light green, carbon from the methyl group. Thermal ellipsoids are shown at the 50% probability level. All hydrogen atoms and minor parts of the disordered lateral chains and methyl groups are omitted for clarity. (B) Fragment of *trans*-**2** crystal packing.

Single crystals of *trans*-**2** were grown from a methanol/THF *cis*/*trans* mixture, and the structure was determined by X-ray crystallography (Figure 3A and Table S1). To the best of our knowledge, the X-ray structure of the related DDSQ **2** hydrochloride salt has not been described to date. Compound *trans*-**2** crystallizes in the rhombohedral crystal lattice with space group *R̅*3. Si(CH_3_)(CH_2_CH_2_CH_2_NH_3_^+^) parts, chloride anion, and some inner core atoms are disordered and refined in two positions each, with site occupation factors equal to 0.75 and 0.25. In addition to that, the position of Si atoms and methyl groups in the crystal structure clearly indicates the *trans*-isomer; the torsion angle between Si0A-C0A-C0A-SiA is 180°.

**Figure 4:**
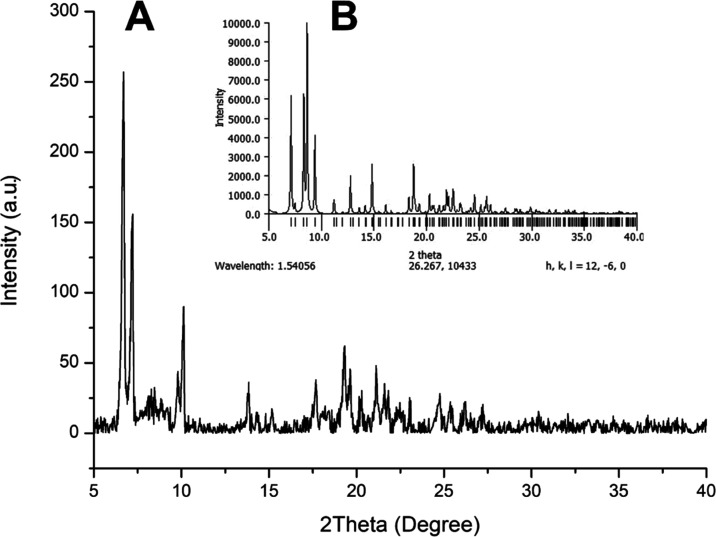
(A) Powder XRD spectrum of *trans*-**2**. (B) Calculated PXRD pattern of *trans*-**2**.

The Si–O bond lengths are in the ranges of 1.59–1.72 Å (average value: 1.64 Å), which follow the already reported DDSQ structures.^16,36,38,48,52^ Because the resulting structure is centrosymmetric, the asymmetric part consists of half a DDSQ molecule and one chloride anion. There are nine molecules in the unit cell; its volume is 1916.1(7) Å^3^. The complex symmetry of the packing of the molecules is caused by the ionic interaction of the chloride anions and the ammonium groups at the ends of the *trans*-**2** chains; statistically, one chloride anion interacts with six ammonium groups. This three-dimensional arrangement of molecules and anions results in one-dimensional channels along the *c*-axis (Figure 3B). According to PLATON’s calculation, the total potential solvent-accessible void is 4277.5 e^–^/Å^3^ (22.3% of unit cell volume). For instance, this solvent-accessible void may correspond to 107 water molecules or ca. 20 small molecules, such as toluene.^55^ The solvent’s atoms could not be identified because it was highly disordered and had a small residual peak. Therefore, SQUEEZE in the PLATON program was performed to remove the highly disordered solvent molecules; mask void content was assigned to 31 MeOH and 24 THF molecules per unit cell. Confirmation that crystals of **2** contain crystallization solvent in the void spaces is provided by thermal gravimetric analysis (TGA) (Figure S14). The TGA graph showed a rapid drop from 46 to 76 °C. Here, the decrease corresponds to the loss of methanol and THF, whose boiling points are 64.7 and 66 °C.^56^ To confirm whether this molecular packing motif is present in the solid, powder X-ray diffraction (PXRD) was measured. It turns out that the diffraction peaks in the calculated pattern,^57^ based on the crystal structure, coincide with the measured one (Figure 4A, B). This can prove the presence of channels for the polycrystalline form, suggesting the potential use of **2** as a sorbent material.

In the final step, amidation reactions were carried out on a *cis*/*trans* mixture, but it was found that the pure *trans*-**2** isomer could be obtained by recrystallization in THF (Figure S11). Amide-DDSQs were prepared by the condensation of **2** with acids in the presence of EDC as a coupling agent and triethylamine as a base. In the first step, a stoichiometric amount of triethylamine was added to **2** dissolved in an appropriate amount of DCM. The mixture was left to stir for about 30 min to neutralize the hydrochloride **2**. The neutralization could be monitored visually; the “milky” mixture becomes transparent when neutralization is over. Next, the mixture was cooled to 0 °C, and acid was slowly added. In the described reaction, methacrylic acid, 4-iodobenzoic acid, and 5-hexynoic acid were used to obtain the amide derivatives of DDSQ, respectively: 3,13-bi(*N*-3-propylmethacrylamide) DDSQ (**3**), 3,13-bi(*N*-3-propyliodobenzamide) DDSQ (**4**), and 3,13-bi(*N*-3-propylhex-5-ynamide) DDSQ (**5**). The routine workup includes washing with saturated NaHCO_3_, water, 0.05 M hydrochloric acid, and brine. The acid is necessary to remove the EDC but must be used at a low concentration due to the sensitivity of the siloxane’s core to hydrolysis. The reaction was efficient for each examined carboxylic acid and enabled the synthesis of designed DDSQ-based systems with two amide-functionalized lateral organic arms. We have selected the acids so that the obtained amide-DDSQs have functional groups that could undergo further modifications, i.e., polymerization, substitution, or azide-alkyne cycloaddition. Derivatives **3**–**5** were obtained in decent yields (74–89%) without additional column purification or recrystallization. All of the obtained products are air-stable solids soluble in THF, DCM, chloroform, hexane, acetonitrile, and toluene and can be synthesized on a multigram scale. All resulting amides were fully characterized using multinuclear NMR (^1^H, ^13^C, and ^29^Si), FT-IR, and MALDI-TOF MS (Figures S15–S32).

^29^Si NMR chemical shifts of **3** were within the expected region for phenyl-substituted closed double-decker silsesquioxane at δ = −17.74 (SiCH_3_), −78.54, −79.55, and −79.67 (Si–O–Si-Ph) ppm for *cis*-**3** and at −17.74 (SiCH_3_), −78.54, and −79.40 ppm for *trans*-**3** isomer (Figure S17). The ^1^H NMR spectrum confirms the presence of methacrylate double bonds at δ = 5.26 and 5.12 ppm (multiplets). A broad peak from the amides group appears at δ = 5.45–5.27 ppm. Moreover, protons from the methacrylate methyl group are localized at δ = 1.76 ppm and three multiplets from methylene hydrogens at δ = 1.76, 1.62–1.48, and 0.71 ppm. The occurrence of two singlets at δ = 0.29 ppm (SiCH_3_) in ^1^H NMR suggests the existence of **3** as a mixture of isomers (Figure S15). Also, the FT-IR spectrum (Figure S18) confirms that **3** was successfully obtained. A broad absorption at 3356 cm^–1^ is due to the stretching vibrations of the NH from the amide group, a band at 1658 cm^–1^ is associated with C=O stretching vibrations, a band at 1622 cm^–1^ with medium intensity is characteristic of stretching vibrations coming from CH=CH_2_ methacrylic group, and the existence of intensive and broad signal at 1133 cm^–1^ proves the occurrence of Si–O–Si bonds.

**Figure 5:**
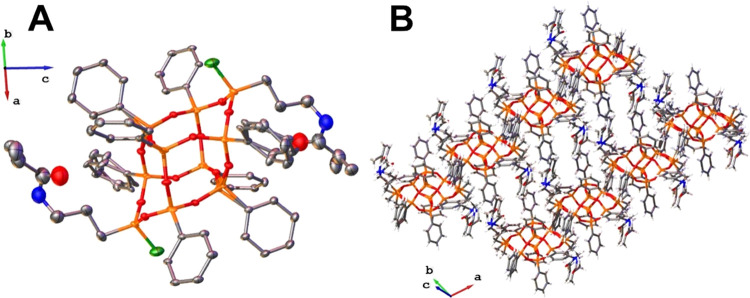
(A) Crystal structure of *trans*-**3**: silver, carbon; orange, silicon; red, oxygen; blue, nitrogen; dark green, chloride; and light green, carbon from the −CH_3_ group. Thermal ellipsoids are shown at the 50% probability level. All hydrogen atoms, THF molecule, minor parts of the disordered lateral chains, and methyl groups are omitted for clarity. (B) Fragment of *trans*-**3** crystal packing.

Furthermore, single crystals of **3** suitable for X-ray diffraction analysis were also obtained (Figure 5 and Table S1). Colorless crystals grew at room temperature from THF-saturated solution as a solvate. **3** crystallizes in triclinic (*P*1̅) space group. Like most *trans-*isomer DDSQ structures,^16,49,53^ the molecule is centrosymmetric, and the asymmetric unit contains half of a molecule. The mutual position of the methyl groups (torsion angle C–Si–Si–C = 180°) clearly indicates that it is a *trans*-isomer (Figure 5A). Lateral side chains were found to be disordered and were refined in two positions each, with site occupation factors equal to 0.62 and 0.38. The molecules do not interact with each other through hydrogen bonds because a THF molecule in the structure is the acceptor of the hydrogen bond from each amide group (Figure 5B). Presumably, the solvent stabilizes the structure and acts as a spacer between the molecules, which we have already observed for other types of DDSQ derivatives.^16^ Obtaining a crystal of the pure *trans*-isomer from a *cis*/*trans* mixture suggests that (i) fractional recrystallization in THF could be an effective separation method and (ii) assumptions about the purity of the isomeric fraction should not be drawn based on the obtained crystal structure; it should be additionally supported by the ^29^Si NMR spectrum dealing with the appropriate number of chemical shifts. Regarding the MALDI-TOF spectrum (Figure S19), the correct mass for the monosubstituted Na^+^ adducts is observed for *m*/*z* = 1425.15, corresponding to the C_64_H_70_N_2_O_16_Si_10_ formula.

In turn, for **4**, ^29^Si NMR signals confirm the existence of a *cis*/*trans* mixture. The presence of *cis*-**4** silicon atoms is observed at δ = −17.70 (*Si*CH_3_), −78.50, −79.32, and −79.67 (Si–O–Si-Ph) ppm and for *trans*-**4** at δ = −17.70 (*Si*CH_3_), −78.50, and −79.51 (Si–O–Si*-*Ph) ppm (Figure S23). The ^1^H NMR spectrum shows that distinctive signals at δ = 7.61 and 7.15 ppm (doublet of doublets) match to the protons of *p*-iodobenzoic groups; at δ = 5.72 ppm (doublet of triplets) comes from the amide group’s protons (NH); the three multiplets at δ = 3.38–3.29, 1.78–1.64, and 0.79 ppm are indicative for methylene’s protons. Since the substrate was a mixture of geometrical isomers, there are two expected singlets at δ = 0.35 ppm (Figure S21). Moreover, absorption bands in the FT-IR spectrum indicate the presence of characteristic groups, i.e., a broad absorption at 3400 cm^–1^ responds to the stretching vibration of the NH group; the intensive band at 1703 cm^–1^ is due to C=O stretching vibrations, 1431 cm^–1^ reveals stretching vibrations of Si–CH_3_, the intensive broad absorption at 1133 cm^–1^ confirms the presence of Si–O vibrations of siloxane core bonds (Figure S24). Additionally, the MALDI-TOF spectrum exhibits an *m*/*z* peak at 1726.33, which corresponds to the monoprotonated adduct of C_70_H_68_I_2_N_2_O_16_Si_10_ (Figure S25).

^29^Si NMR spectrum of **5** also indicates a mixture of isomers: *cis*-**5**: δ = −17.74 (*Si*CH_3_), −78.55, −79.26, and −79.75 (Si–O–Si-Ph) ppm; *trans*-**5**: δ = −17.74 (*Si*CH_3_), −78.55, and −79.59 (Si–O–Si-Ph) ppm (Figure S29). In the ^1^H NMR, the occurrence of the following groups of protons was confirmed: the amide group (NH) at δ = 5.02 ppm (doublet of triplets); the acetylene group at δ = 1.88 pm (multiplet), and six signals from methylene’s protons at δ = 3.15–3.03, 2.12, 2.03–1.94, 1.68, 1.60–1.46, and 0.70 ppm. Like in the case of **3** and **4**, the signal of SiCH_3_ is doubled due to the presence of two isomers (Figure S27). In addition, the FTR-IR spectrum indicates the presence of NH amide at 3303 cm^–1^, carbonyl group (C=O) at 1649 cm^–1^, methyl groups attached to lateral silicon atoms at 1431 cm^–1^, and finally, the absorption band matching the asymmetric stretching of Si–O–Si bonds exhibits at 1133 cm^–1^ (Figure S30). In turn, the MALDI-TOF spectrum also confirms obtaining the derivative with the assumed structure due to the *m*/*z* peak at 1477.15, which corresponds to the monosodium adduct of C_68_H_74_N_2_O_16_Si_10_ (Figure S31).

## Conclusions

A series of new bifunctionalized DDSQ derivatives with alkene, alkyne, and *p*-iodophenyl substituents were synthesized with conventional amidation reactions with high yields. A new procedure was presented, considering the siloxane core’s susceptibility to hydrolysis under acidic conditions. The use of this approach opens the way to the synthesis of new DDSQ derivatives and materials using BOC-blocked amines. Moreover, according to our best knowledge, we present for the first time a method for the synthesis of DDSQ amine hydrochloride salt, which has better stability than the amine derivative. Their interesting reactivity can be used in some specific functionalization methods, which are unapproachable for amine groups, i.e., aminochlorination of maleimides,^57^ amide synthesis using aldehydes,^58^ ring-opening polymerization,^59,60^ Mannich reaction,^61^ Chichibabin pyridine synthesis,^62^ 4-amino imidazole synthesis,^63^ or the synthesis of pyrrolin-4-ones.^64^

Furthermore, the crystal structure of the hydrochloride salt *trans-***2** has been resolved and refined. The crystal structure model indicates the presence of channels in the network, which the PXRD and TGA-DTA analysis also confirmed. We also observed an unprecedented isomerization process of the DDSQ derivative. In addition, we propose its possible reason and a method for separating the pure *trans*-isomer. We believe our findings would advance investigations about new materials based on intriguing, inorganic–organic DDSQ compounds.

## Experimental Section

All of the reactions and operations that required an inert atmosphere of N_2_ or Ar were performed using a standard Schlenk apparatus and vacuum line techniques. Solvents for the synthesis (THF, toluene) were purified using Solvent Purification Systems (Inert, PureSolv EN 1-7 Base). Catalyst species were removed after reaction by filtration through a Celite 545 (Sigma-Aldrich) pad. Solvents for standard workup (methanol, hexane, acetone) were purchased from VWR International and ChemPur and were used as received. All of the chemicals were obtained from commercial sources and used without further purification: TetraSilanolPhenyl POSS (Hybrid Plastics Inc.), dichloromethylsilane (>97% Sigma-Aldrich), Karstedt Catalyst (platinum(0)-1,3-divinyl-1,1,3,3-tetramethyldisiloxane complex solution) (in xylene, Pt ∼ 2%, Sigma-Aldrich), *tert*-butyl allylcarbamate (>97%, Fluorochem), oxalyl chloride (>98%, Sigma-Aldrich), methacrylic acid (>99%, Aldrich), 1-(3-dimethylaminopropyl)-3-ethylcarbodiimide (EDC) (>97% abcr GmbH), triethylamine (Et_3_N) (>99%, Sigma-Aldrich), 4-iodobenzoic acid (>97%, Thermo Scientific), 5-hexynoic acid (>96%, TCI Chemicals), sodium bicarbonate (≥99%, Chempur), hydrochloric acid (35–38%, Chempur), sodium chloride (>99.5%, Chempur), and magnesium sulfate (anhydrous, >99%, Chempur). ^1^H, ^13^C NMR, and ^29^Si NMR spectra were recorded with a Bruker Avance III 500 MHz spectrometer or with a Jeol JNM-ECZ500R 500 MHz spectrometer. Chemical shifts are reported in parts per million (ppm) downfield from tetramethylsilane and are referenced to residual peaks of deuterated NMR solvents. Chemical shift values for ^29^Si{^1^H} NMR spectra were referenced to TMS or DSS. Assignments are based on COSY and HSQC correlation experiments. Coupling constants (*J*) are reported in Hz. Standard abbreviations s, d, t, q, and m refer to singlet, doublet, triplet, quartet, and multiplet. High-resolution mass spectra were recorded using a JMS-S3000 SpiralTOF-plus 2.0 spectrometer with dithranol as a MALDI matrix. FT-IR spectra were recorded on a Bruker Vertex 70 FT-IR spectrometer in the transmission mode in the 4000–400 cm^–1^ range. The sample chamber was continuously flushed with N_2_. The spectra were recorded using KBr pellets. Optical-grade, random cuttings of KBr were ground with 1.0 wt% of the sample to be analyzed and pressed as KBr pellets. Elemental analyses (C, H) were performed using a Vario EL III element analyzer (Hanau, Germany). Single-crystal X-ray diffraction data for **1–3** were collected on a XtaLAB Synergy R, DW system, HyPix-Arc 150 with the scan technique at 100 K. The data collection and processing utilized CrysAlis suite of programs. The structure was solved by direct methods using the SHELXL-2018 program and refined by full-matrix least squares on *F*^2^. All nonhydrogen atoms were refined with anisotropic displacement parameters, and hydrogen atoms were placed at their calculated positions. Crystallographic data for the structural analysis has been deposited with the Cambridge Crystallographic Data Centre, Nos. 2211995 (**1**), 2209518 (**2**), and 2217825 (**3**). Copies of this information may be obtained free of charge from: The Director, CCDC, 12 Union Road, Cambridge, CB2 1EZ, U.K. Fax: +44(1223)336-033, Email: deposit@ccdc.cam.ac.uk, or www: www.ccdc.cam.ac.uk.

### Synthesis of 3,13-Bis(3-*tert*-butoxycarbonylaminopropyl) DDSQ (**1**)

Note: **DDSQ-Me**_**2**_**H**_**2**_ was synthesized according to the literature procedure.^16^

**DDSQ-Me**_**2**_**H**_**2**_ (3 g, 0.26 mmol) was added to anhydrous toluene (200 ml) under a nitrogen atmosphere, heated to 40 °C, and stirred until the substrate dissolved completely. Then Karstedt catalyst (20 μL, 0.896 μmol) was added. After stirring for 30 min, *tert*-butyl-*N*-allylcarbamate (1.022 g, 1.1 mL, 6.5 mmol) was added, and the mixture was heated to 60 °C. The reaction was continuously stirred and heated for 24 h, after which it was cooled to RT. The solution was passed through a pad of Celite and concentrated on a rotary evaporator giving yellow oil. *Separation:* the precipitate (2.8 g, pure *trans*-isomer) was obtained by three times repeated crystallization from cold methanol (100 mL). After filtration, the precipitate was collected and dried in vacuum. The product was obtained as a white solid with a yield of 71% (2.71 g, 1.85 mmol). ^1^H NMR (500 MHz, CDCl_3_) δ 7.62–7.13 (m, -Ph, 40H), 4.25–4.21 (s, CH__2__NHCO, 2H), 3.00–2.93 (m, CH_2_CH_2_CH__2__NH, 4H), 1.54–1.49 (m, CH_2_CH_2_CH__2__NH, 4H), 1.40–1.36 (s, COO(CH_3_)_3_, 18H), 0.70–0.66 (m, CH_2_CH_2_CH__2__NH,4H), 0.3–0.27 (s, Si–CH_3_, 6H). ^13^C NMR (151 MHz, CDCl_3_) δ 156.71 (C=O), 134.86, 134.74, 132.74, 131.78, 131.29, 128.71, 128.59 (Ph), 79.72 (C(CH_3_)_3_), 43.97 (CH_2_NH), 29.25 (C(CH_3_)_3_), 24.17 (SiCH_2_CH_2_CH_2_NH), 14.69 (SiCH_2_CH_2_CH_2_NH), 0.00 (Si*C*H_3_).^29^Si NMR (119 MHz, CDCl_3_) (pure *trans*-isomer) δ −17.79 (SiCH_3_), −78.53, 79.52 (Si–O–Si-Ph). FT-IR (KBr, νmax/cm^–1^): 3440 (br, N–H), 3073, 3052, 3027 (C–H_arom_.), 2979, 2932 (C–H_aliph_.), 1698 (C=O), 1595 (C=C_arom_), 1431 (Si–CH_3_), 1132 (br, Si–O–Si). MALDI-TOF MS (*m*/*z*): calculated for C_66_H_78_O_18_N_2_Si_10_ [M + Na]^+^: 1489.28; found: 1489.28. Elemental analyses calculated for C_66_H_78_O_18_N_2_Si_10_: C, 53.99; H, 5.36; N, 1.91; Si, 19.13; found: C, 53.87; H, 5.40; N, 1.92; Si, 19.12. Crystal data for C_66_H_78_O_18_N_2_Si_10_ (M = 1468.20 g/mol): monoclinic, space group *P*21/*c* (no. 14), *a* = 11.027(4) Å, *b* = 16.624(7) Å, *c* = 19.817(3) Å, β = 95.910(10)°, *V* = 3613(2) Å^3^, *Z* = 2, *T* = 99.99(10) K, μ(Cu Kα) = 2.297 mm^–1^, Dcalc = 1.349 g/cm^3^, 36 442 reflections measured (6.956 ≤ 2Θ ≤ 146.786°), 6989 unique (*R*_int_ = 0.0305, *R*_sigma_ = 0.0283), which were used in all calculations. The final *R*_1_ was 0.0349 (*I* > 2σ(*I*)) and w*R*_2_ was 0.0891 (all data).

### Synthesis of 3,13-Bi(aminopropyl) DDSQ Hydrochloride (**2**)

#### Deprotection of **1**

To a cold (0 °C) solution of **1** (347 mg, 0.236 mmol) in methanol (20 mL), stirring under dinitrogen flow, oxalyl chloride (320 mg, 217 μL, 2.53 mmol) was added slowly. The Schlenk vessel was closed with a septum with a needle—to allow the evolution of CO and CO_2_ during the reaction. The reaction mixture was stirred at room temperature for 24 h. After the reaction was completed (the “milky” solution changes to transparent), the solvent was evaporated. The resulting solid was washed three times with distilled water and dried in vacuo. The product was obtained as a white solid with a yield of 75% (238 mg, 0.178 mmol). ^1^H NMR (500 MHz, MeOD) δ 7.60–7.12 (m, -Ph, 40H), 2.83 (t, *J* = 7.4 Hz, CH_2_NH_3_^+^, 4H), 1.83–1.66 (m, SiCH_2_CH_2_CH_2_, 4H), 0.91–0.77 (m, SiCH_2_CH_2_CH_2_, 4H), 0.43 (d, *J* = 1.9 Hz, SiCH_3_, 6H). ^13^C NMR (126 MHz, MeOD) δ 134.95, 134.92, 132.60, 132.00, 129.13, 129.00 (Ph), 43.14 (*C*H_2_NH_3_^+^), 22.25 (SiCH_2_CH_2_CH_2_), 14.32 (SiCH_2_CH_2_CH_2_), −0.99 (SiCH_3_). ^29^Si NMR (99 MHz, MeOD) (*cis*/*trans*-isomers) δ −18.15 (SiCH_3_), −78.12, −78.90, −79.11, −79.32. (Si–O–Si-Ph). ^29^Si NMR (99 MHz, MeOD) (*trans*-isomer) δ −18.19 (*Si*CH_3_), −78.13, −79.11 (Si–O–Si-Ph). FT-IR (KBr, νmax/cm^–1^): 3435 (br, N–H), 3073, 3051, 3007 (C–H_arom_.), 2925 (C–H_aliph_.), 1600 (N–H), 1510 (N–H), 1594 (C=C_arom_), 1430 (Si–CH_3_), 1132 (br, Si–O–Si). Crystal data for C_70.11_Cl_0.5_H_99.11_N_2_O_20.11_Si_10_ (*M* = 1594.73 g/mol): trigonal, space group *R*3̅ (no. 148), *a* = 40.8750(10) Å, *c* = 13.253 Å, *V* = 19176.1(9) Å^3^, *Z* = 9, *T* = 100.00(10) K, μ(Cu Kα) = 2.142 mm^–1^, Dcalc = 1.243 g/cm^3^, 42 261 reflections measured (8.334 ≤ 2Θ ≤ 147.286°), 8274 unique (*R*_int_ = 0.0499, *R*_sigma_ = 0.0319), which were used in all calculations. The final *R*_1_ was 0.1023 (*I* > 2σ(*I*)) and w**R**_2_ was 0.3205 (all data).

### Synthesis of 3,13-Bi(*N*-3-propylmethacrylamide) DDSQ (**3**)

**2** (1.15 g, 0.858 mmol) and dry dichloromethane (100 mL) were charged in a two-necked flask equipped under dinitrogen flow. To the stirred, “milky” suspension, Et_3_N (0.26 g, 359 μL, 2.57 mmol) was added dropwise. The mixture was allowed to stir for half an hour (at this time, the “milky” mixture becomes transparent) and cooled to 0 °C. Methacrylic acid (221 mg, 217 μL, 2.57 mmol) and EDC (508 mg, 487 μL, 2.57 mmol) were added dropwise to the cold solution. The reaction mixture was allowed to warm to room temperature and left for stirring for 24 h. The reaction mixture was washed with aqueous, saturated sodium bicarbonate solution, distilled water, 0.05 M hydrochloric acid solution, and brine. The organic layer was dried over MgSO_4_ and concentrated using a vacuum evaporator. The product was obtained as a white solid after drying in vacuum with a yield of 74% (891 mg, 0.635 mmol). ^1^H NMR (500 MHz, CDCl_3_) δ 7.56–7.10 (m, −Ph, 40H), 5.45–5.27 (m, −NH, 2H), 5.12 (m, C(CH_3_)=CH_2_, 2H), 3.16 (m, C(CH_3_)=CH_2_, 2H), 1.76 (m, SiCH_2_CH_2_CH_2_, 4H), 1.62–1.48 (m, SiCH_2_CH_2_CH_2_, 4H), 0.71 (m, SiCH_2_CH_2_CH_2_, 4H), 0.29 (s, SiCH_3_, 6H). ^13^C NMR (126 MHz, CDCl_3_) δ 168.28 (C=O), 140.09 (C(CH_3_)=CH_2_), 133.98, 133.89, 131.78, 130.89, 130.52, 127.91, 127.80 (Ph) 118.98 (C(CH_3_)=CH_2_), 41.96 (SiCH_2_CH_2_CH_2_), 22.95 (SiCH_2_CH_2_CH_2_), 18.56 (C(CH_3_)=CH_2_), 13.90 (SiCH_2_CH_2_CH_2_), −0.80 (SiCH_3_). ^29^Si NMR (99 MHz, CDCl_3_) (cis/trans-isomers) δ −17.74 (SiCH_3_), −78.54, −79.55, −79.40, −79.67 (Si–O–Si-Ph). FT-IR (KBr, νmax/cm^–1^): 3356 (br, N–H), 3074, 3054, 3028, (C–H_arom_.), 2959, 2926 (C–H_aliph_.), 1658 (C=O), 1622 (C=C), 1595 (C=C_arom_), 1533 (C–N), 1431 (Si–CH_3_), 1133 (br, Si–O–Si). MALDI-TOF MS (*m*/*z*): calculated for C_64_H_70_N_2_O_16_Si_10_ [M + Na]^+^: 1425.23; found: 1425.15. Crystal data for C_72_H_86_N_2_O_18_Si_10_ (*M* = 1546.17 g/mol): triclinic, space group *P*1̅ (no. 2), *a* = 9.970(8) Å, *b* = 13.052(8) Å, *c* = 15.935(8) Å, α = 102.520(10)°, β = 91.88(3)°, γ = 112.30(9)°, *V* = 1858(2) Å^3^, *Z* = 1, *T* = 100.2(8) K, μ(Cu Kα) = 2.263 mm^–1^, Dcalc = 1.382 g/cm^3^, 21 304 reflections measured (5.728 ≤ 2Θ ≤ 146.738°), 6993 unique (*R*_int_ = 0.0389, *R*_sigma_ = 0.0276), which were used in all calculations. The final *R*_1_ was 0.0966 (*I* > 2σ(*I*)) and *w*R**_2_ was 0.2878 (all data).

### Synthesis of 3,13-Bi(*N*-3-propyliodobenzamide) DDSQ (**4**)

The synthesis of **4** proceeded analogically to that of **3**, but instead of methacrylic acid, 4-iodobenzoic acid (116 mg, 0.468 mmol) was used. The product was obtained as a white solid after drying in vacuum with a yield of 74% (240 mg, 0.139 mmol). ^1^H NMR (500 MHz, CDCl_3_) δ 7.61 (dd, *J* = 10.1, 7.7 Hz, C=H_arom_, 4H), 7.56–7.18 (m, -Ph, 40H), 7.15 (dd, *J* = 8.9, 7.0 Hz, C=H_arom_, 4H), 5.72 (dt, *J* = 19.6, 5.8 Hz, NH, 2H), 3.38–3.29 (m, SiCH_2_CH_2_CH_2_NH, 4H), 1.78–1.64 (m, SiCH_2_CH_2_CH_2_NH, 4H), 0.79 (m, SiCH_2_CH_2_CH_2_NH, 4H), 0.35 (d, *J* = 7.0 Hz, SiCH_3_, 6H). ^13^C NMR (126 MHz, CDCl_3_) δ 167.16 (C=O), 138.25 (Ph), 134.70, 134.69, 134.62, 134.56, 132.39, 132.36, 131.51, 131.45, 131.42, 131.33, 131.29, 131.20, 129.08, 98.68 (Ph), 42.98 (SiCH_2_CH_2_CH_2_NH), 23.65 (SiCH_2_CH_2_CH_2_NH), 14.55 (SiCH_2_CH_2_CH_2_), 0.00 (SiCH_3_). ^29^Si NMR (99 MHz, CDCl_3_) (*cis*- and *trans*-isomers) δ −17.70 (SiCH_3_), −78.50, −79.32, −79.51, −79.67 (Si–O–Si-Ph). FT-IR (KBr, νmax/cm^–1^): 3400 (br, N–H), 3073, 3051, 3028 (C–H_arom._), 2962, 2932, 2869, 2824 (C–H_aliph.),_ 1703 (C=O), 1651 (C=C), 1588 (C=C_arom_), 1532 (C–N), 1431 (Si–CH_3_), 1133 (br, Si–O–Si). MALDI-TOF MS (*m*/*z*): calculated for C_70_H_68_I_2_N_2_O_16_Si_10_ [M + H]^+^: 1726.03; found: 1726.33. Elemental analyses calculated for C_56_H_64_Cl_2_N_2_O_14_Si_10_: C, 50.16; H, 4.81; N, 2.09; Si, 20.95; found: C, 50.08; H, 4.80; N, 1.98; Si, 21.04.

### Synthesis of 3,13-Bi(*N*-3-propylhex-5-ynamide) DDSQ (**5**)

The synthesis of **5** proceeded analogically to that of **3**, but instead of methacrylic acid, 5-hexynoic acid (237 mg, 233 μL, 2.114 mmol) was used. After vacuum drying, **5** was obtained as a yellow solid (1.01 g, 0.693 mmol, 82% yield). ^1^H NMR (500 MHz, CDCl_3_) δ 7.83–7.04 (m, −Ph, 40H), 5.02 (dt, *J* = 17.5, 5.9 Hz, −NH, 2H), 3.15–3.03 (m, SiCH_2_CH_2_CH_2_, 4H), 2.12 (tdd, *J* = 7.0, 4.6, 2.7 Hz, NH(CO)CH_2_, 4H), 2.03–1.94 (m, CH_2_C≡CH, 4H), 1.88 (m, CH_2_C≡CH, 2H), 1.68 (q, *J* = 7.1 Hz, COCH_2_CH_2_CH_2_, 4H), 1.60–1.46 (m, SiCH_2_CH_2_CH_2_, 4H), 0.70 (ddd, J = 10.5, 5.8, 2.1 Hz, m, SiCH_2_CH_2_CH_2_, 4H), 0.36–0.26 (d, SiCH_3_, 6H).^13^C NMR (126 MHz, CDCl_3_) δ 172.70 (C=O), 135.01, 134.92, 134.82, 134.69, 132.59, 131.41, 128.77, 128.65, 128.54 (Ph), 84.39 (CH_2_C≡CH), 69.84 (CH_2_C≡CH), 42.60 (CH_2_NH(CO)), 35.74 (CH_2_C≡CH), 24.88 ((CO)CH_2_CH_2_CH_2_), 23.78 (SiCH_2_*C*H_2_CH_2_), 18.63 ((CO)CH_2_CH_2_CH_2_), 14.67 (SiCH_2_CH_2_CH_2_), 1.84 (Si*C*H_3_).^29^Si NMR (99 MHz, CDCl_3_) (*cis*/*trans*-isomers): δ −17.74 (SiCH_3_), −78.55, −79.26, −79.59, −79.75 (Si–O–Si-Ph). FT-IR (KBr, νmax/cm^–1^): 3303 (br, N–H), 3074, 3052, 3028 (C–H_arom._), 2962, 2933 (C–H_aliph.),_ 1649 (C=O), 1622 (C=C), 1595 (C=C_arom_), 1552 (C–N), 1431 (Si–CH_3_), 1133 (br, Si–O–Si). MALDI-TOF MS (*m*/*z*): calculated for C_68_H_74_N_2_O_16_Si_10_ [M + Na]^+^: 1477.26; found: 1477.15. Elemental analyses calculated for C_68_H_74_N_2_O_16_Si_10_: C, 56.09; H, 5.12; N, 1.92; Si, 19.29; found: C, 56.02; H, 5.20; N, 1.88; Si, 19.29.

## Supporting Information Available

NMR (^1^H, ^13^C, ^29^Si), FT-IR spectra, MALDI-TOF MS, and crystallographic refinement details (CIF and check CIF files) (PDF)
