# Supplementary material for: Unprecedented Route to Amide-Functionalized Double-Decker Silsesquioxanes Using Carboxylic Acid Derivatives and a Hydrochloride Salt of Aminopropyl-DDSQ
Source: Inorg Chem. 2023 Mar 29;62(14):5520–30. doi: 10.1021/acs.inorgchem.2c04546 (PMC10091418; doi:10.1021/acs.inorgchem.2c04546)
Supplement: Supplementary file 1 — ic2c04546_si_001.pdf [file ic2c04546_si_001.pdf]

Supporting Materials

Unprecedented Route to Amide-Functionalized Double-Decker Silsesquioxanes Using Carboxylic Acid Derivatives and Hydrochloride Salt of Aminopropyl-DDSQ

Anna Władyczyn[a] and Łukasz John*,[a]

[a] Faculty of Chemistry, University of Wrocław, 14 F. Joliot-Curie, 50-383 Wrocław, Poland

*Corresponding author: Professor Łukasz John, Ph.D., D.Sc.; e-mail: lukasz.john@uwr.edu.pl

Table of Contents

Figure S1. ^{1}H NMR (500 MHz, $CDCl_3$, 300 K) spectrum of **1**. 3

Figure S2. ^{13}C NMR (151 MHz, CDCl3, 300 K) spectrum of **1**. 3

Figure S3. ^{29}Si NMR (119 MHz, $CDCl_3$) spectrum of *trans*-**1**. 4

Figure S4. ^{29}Si NMR(119 MHz, CDCl3) spectrum of *cis*-**1**. 4

Figure S5. FT-IR (KBr pellet) spectrum of **1**. 5

Figure S6. MALDI-TOF MS spectrum of **1**. 5

Figure S7. TG-DTA diagram of **1** 6

Figure S8. ^{1}H NMR (500 MHz, MeOD, 300 K) spectrum of **2**. 7

Figure S9. ^{13}C NMR (126 MHz, MeOD, 300 K) spectrum of **2**. 7

Figure S10. ^{29}Si NMR (99 MHz, MeOD) spectrum of *cis*-**2** and *trans*-**2**. 8

Figure S11. ^{29}Si NMR (99 MHz, MeOD) spectrum of *trans*-**2** 8

Figure S12. FT-IR (KBr pellet) spectrum of **2** 9

Figure S13. MALDI-TOF MS spectrum of **2** 9

Figure S14. TGA-DTA diagram of **2**. 10

Figure S15. ^{1}H NMR (500 MHz, $CDCl_3$, 300 K) spectrum of **3**. 10

Figure S16. ^{13}C NMR (126 MHz, $CDCl_3$, 300 K) spectrum of **3** 11

Figure S17. ^{29}Si NMR (99 MHz, $CDCl_3$) spectrum of *cis*-**3**/*trans*-**3** 11

Figure S18. FT-IR (KBr pellet) spectrum of **3**. 12

Figure S19. MALDI-TOF MS spectrum of **3**. 12

Figure S20. TG-DTA diagram of **3**. 13

Figure S21. ^{1}H NMR (500 MHz, $CDCl_3$, 300 K) spectrum of **4**. 13

Figure S22. ^{13}C NMR (126 MHz, $CDCl_3$, 300 K) spectrum of **4** 14

Figure S23. ^{29}Si NMR (99 MHz, $CDCl_3$, 300K) spectrum of **4** 14

Figure S24. FT-IR (KBr pellet) spectrum of **4**. 15

Figure S25. MALDI-TOF MS spectrum of **4**. 15

Figure S26. TG-DTA diagram of **4**. 16

Figure S27. ^{1}H NMR (500 MHz, $CDCl_3$, 300 K) spectrum of **5**. 17

Figure S28. ^{13}C NMR (126 MHz, $CDCl_3$, 300K) spectrum of **5**. 17

Figure S29. ^{29}Si NMR (99 MHz, $CDCl_3$) spectrum of **5**. 18

Figure S30. FT-IR (KBr pellet) spectrum of **5**. 18

Figure S31. MALDI-TOF MS spectrum of **5**. 19

Figure S32. TG-DTA diagram of **5**. 19

Table S1. Crystal data and structure refinement parameters for **1**, **2** and **3**. 20

Figure S33. Crystal structure of dimethyl oxalate 21

Table S2. Crystal data and structure refinement parameters for dimethyl oxalate 21

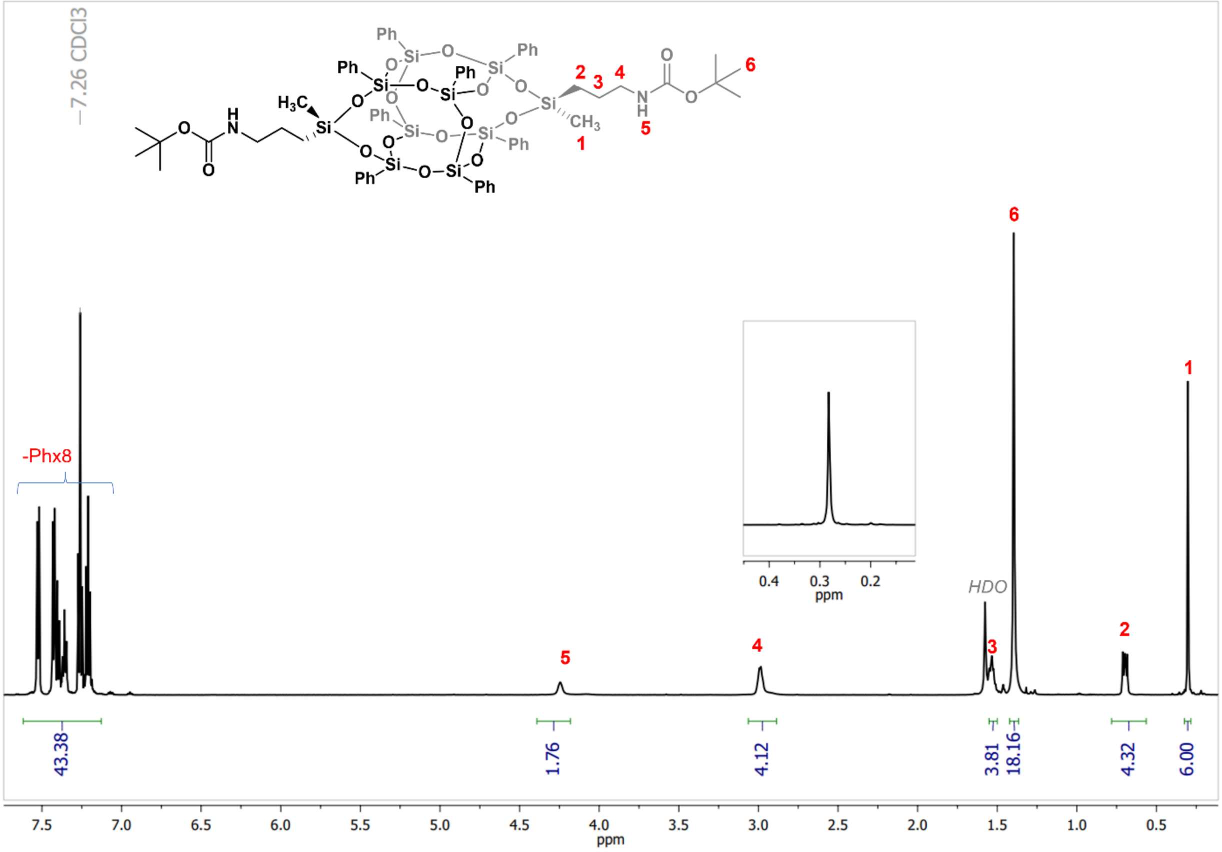

Figure S1. ^{1}H NMR (500 MHz, $CDCl_3$, 300 K) spectrum of **1**.

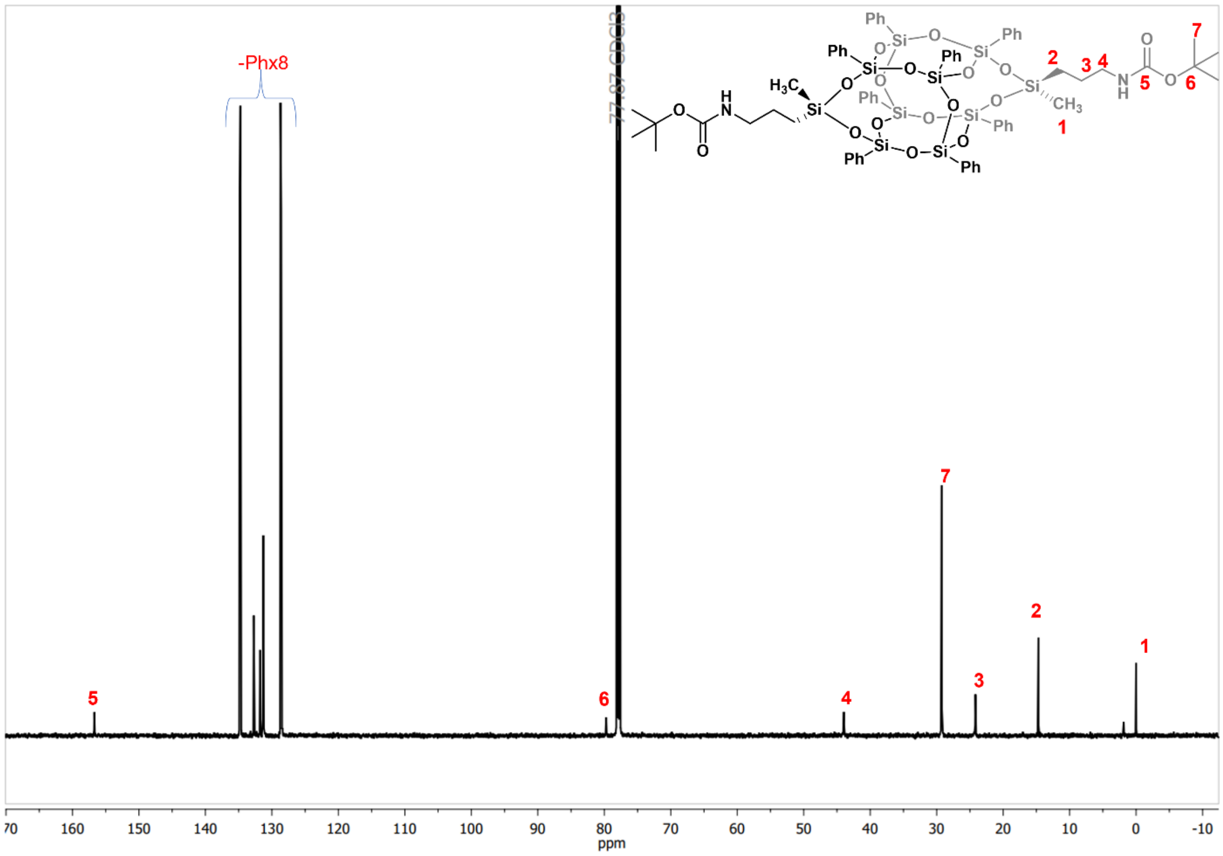

Figure S2. ^{13}C NMR (151 MHz, CDCl3, 300 K) spectrum of **1**.

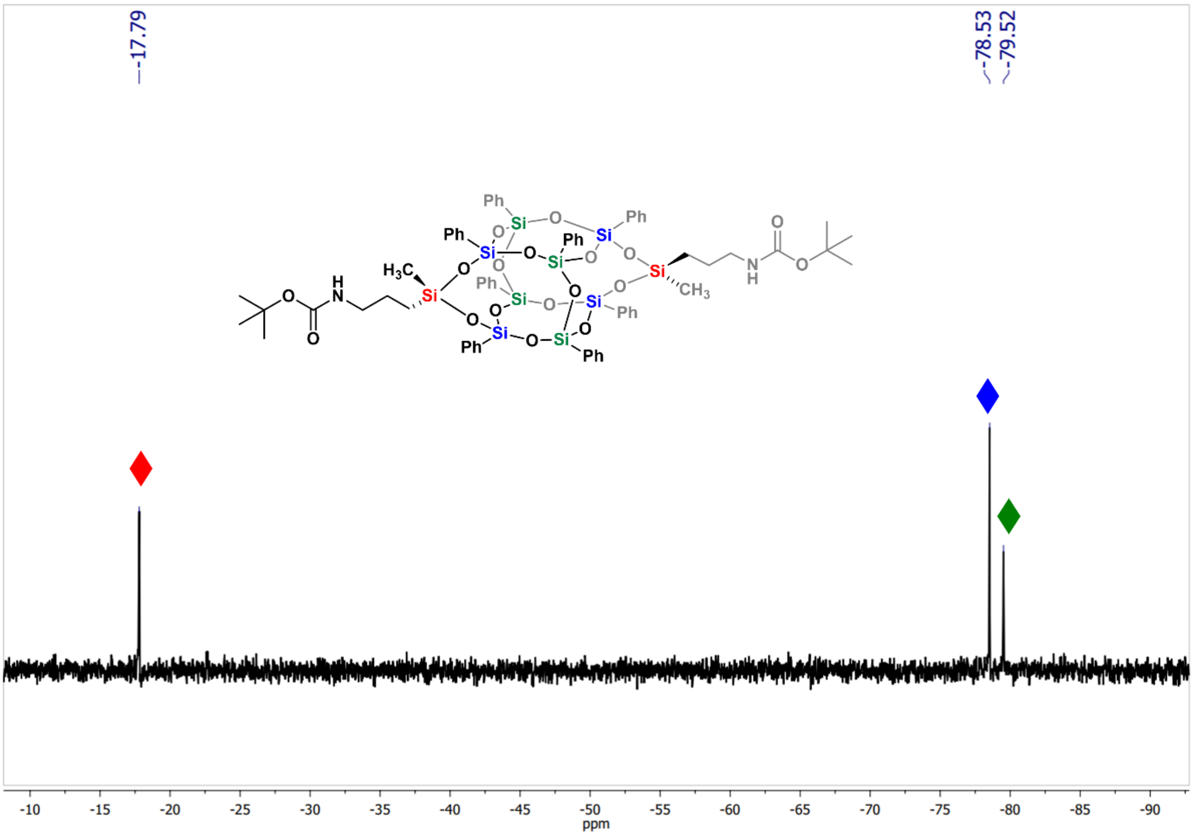

Figure S3. ^{29}Si NMR (119 MHz, $CDCl_3$) spectrum of *trans*-**1**.

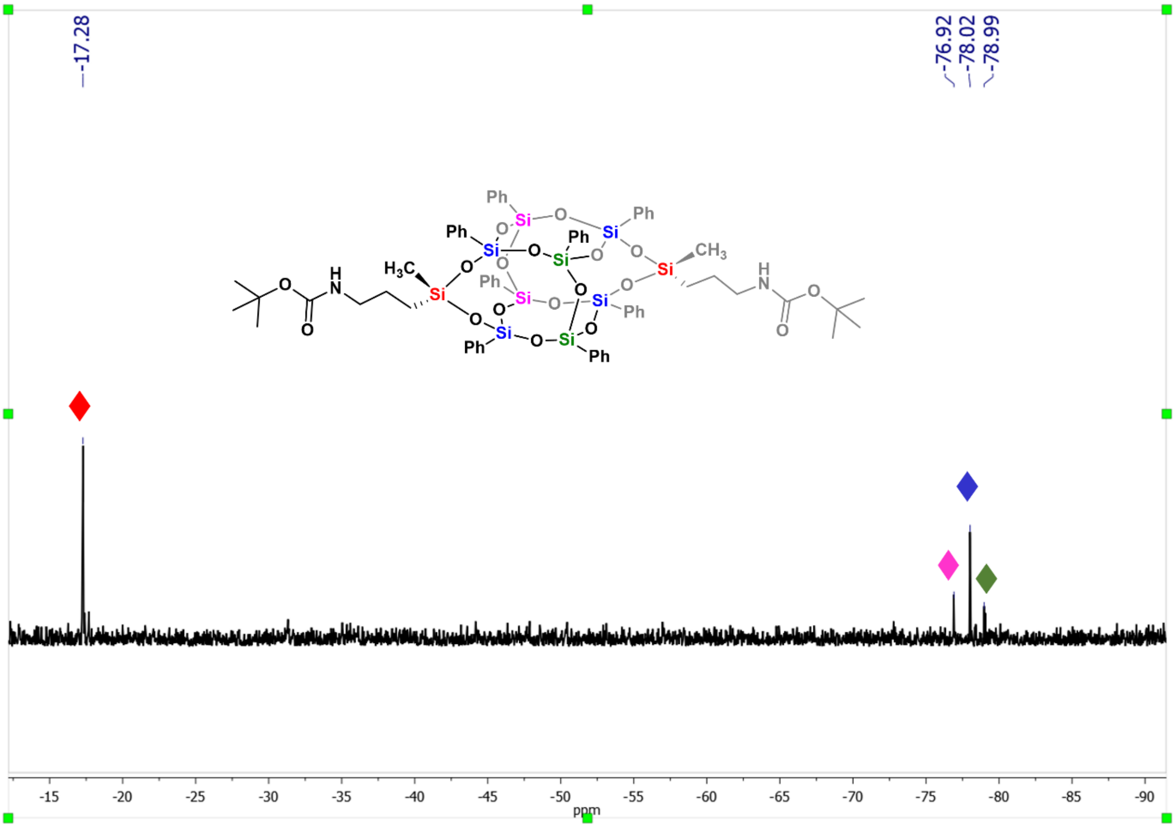

Figure S4. ^{29}Si NMR(119 MHz, CDCl3) spectrum of *cis*-**1**.

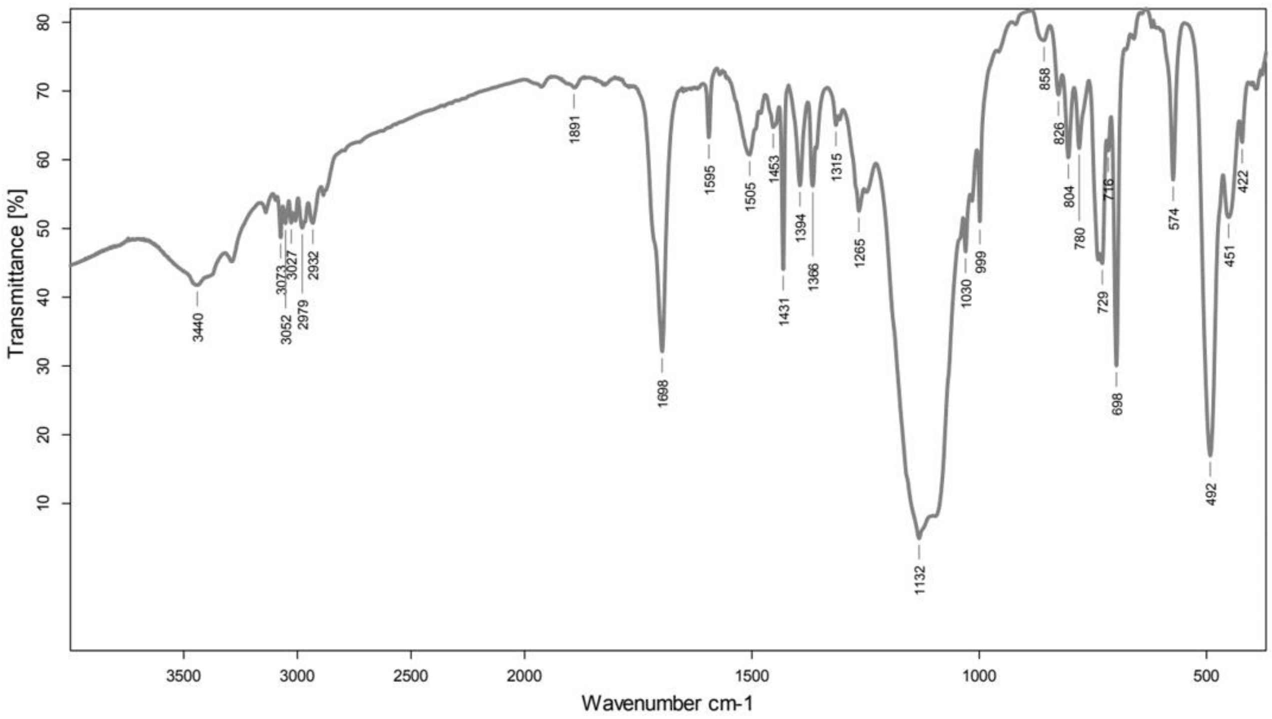

Figure S5. FT-IR (KBr pellet) spectrum of **1**.

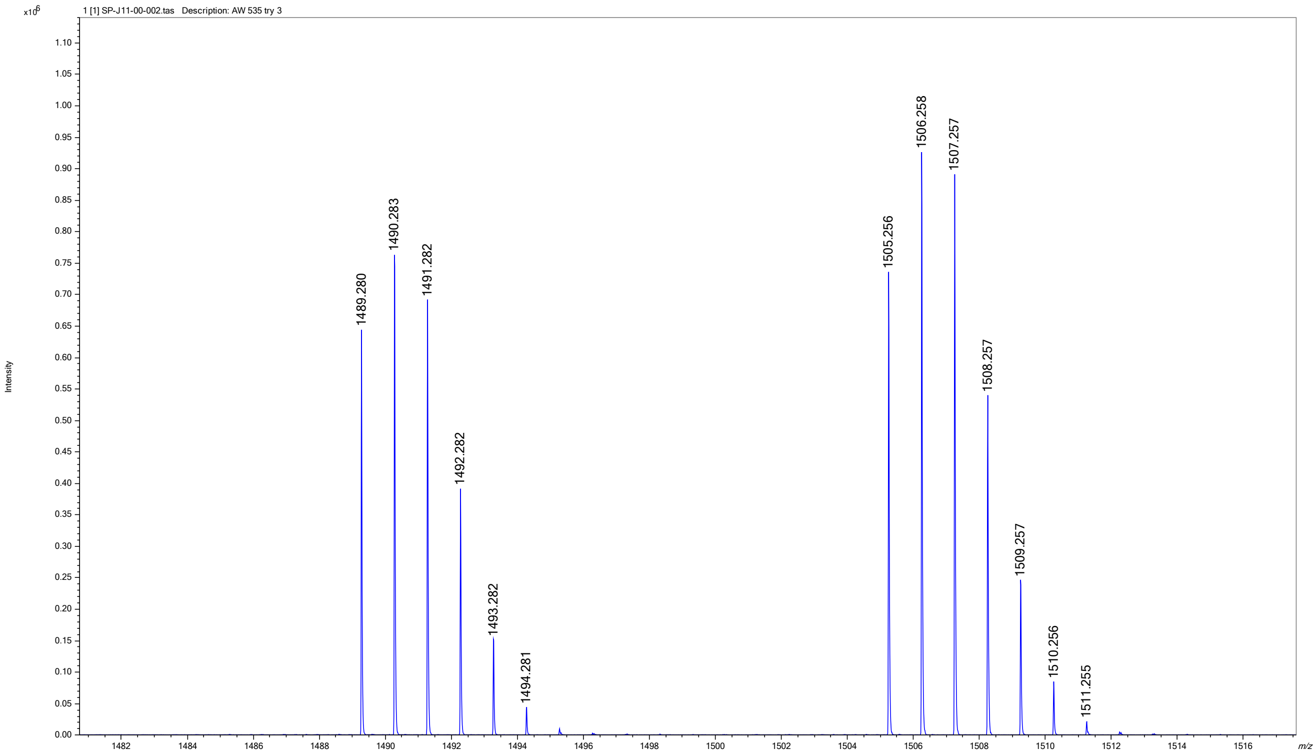

Figure S6. MALDI-TOF MS spectrum of **1**.

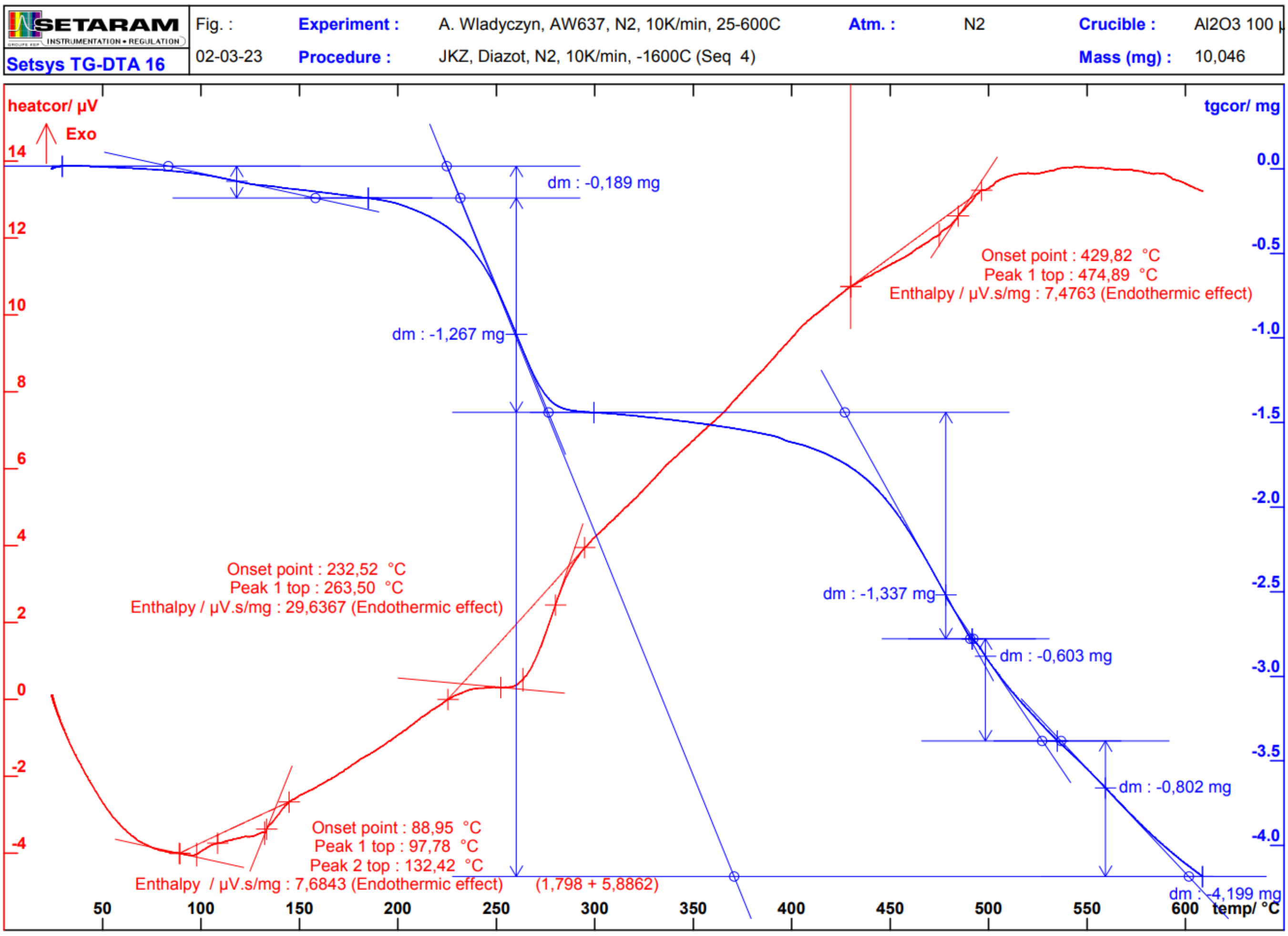

Figure S7. TG-DTA diagram of **1**. Heated 10 K/min under N_2 flow.

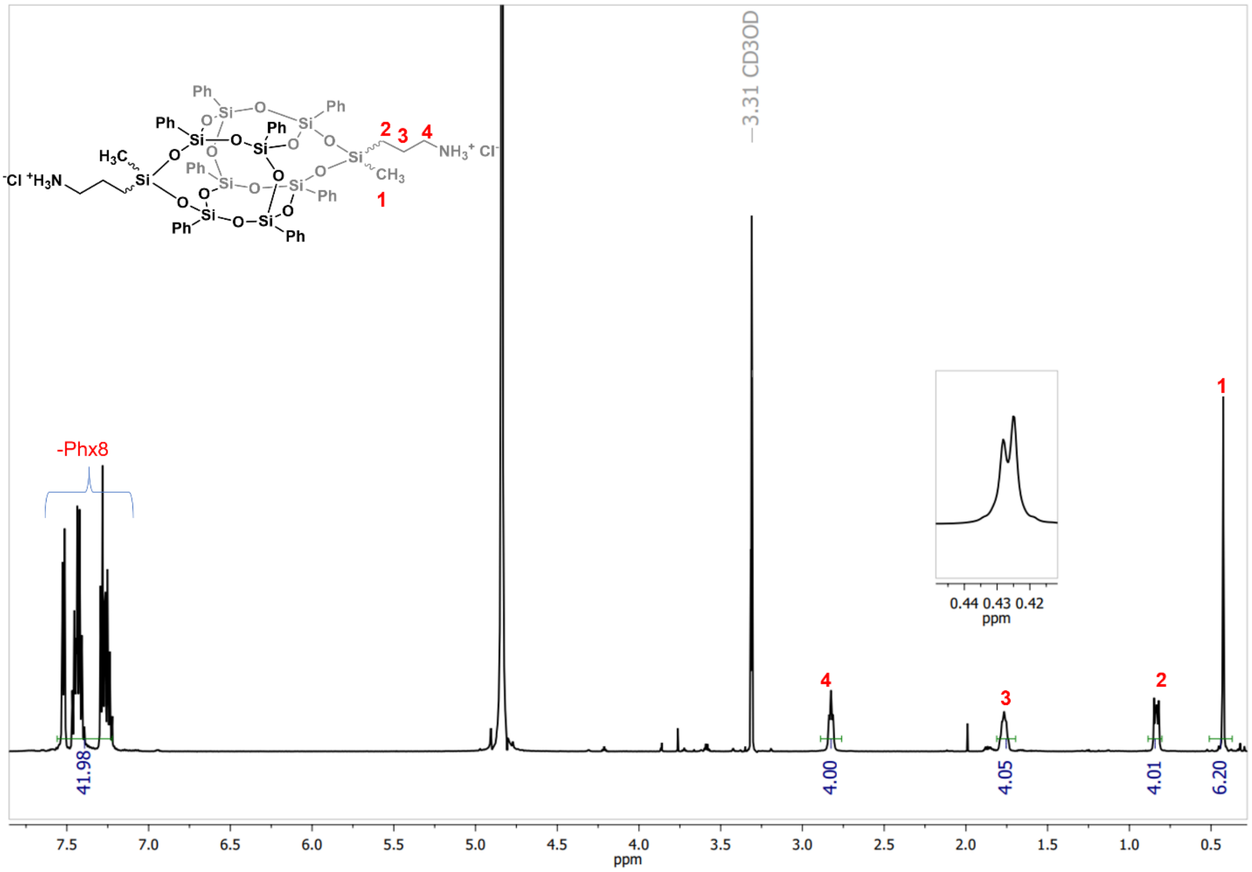

Figure S8. ^{1}H NMR (500 MHz, MeOD, 300 K) spectrum of **2**.

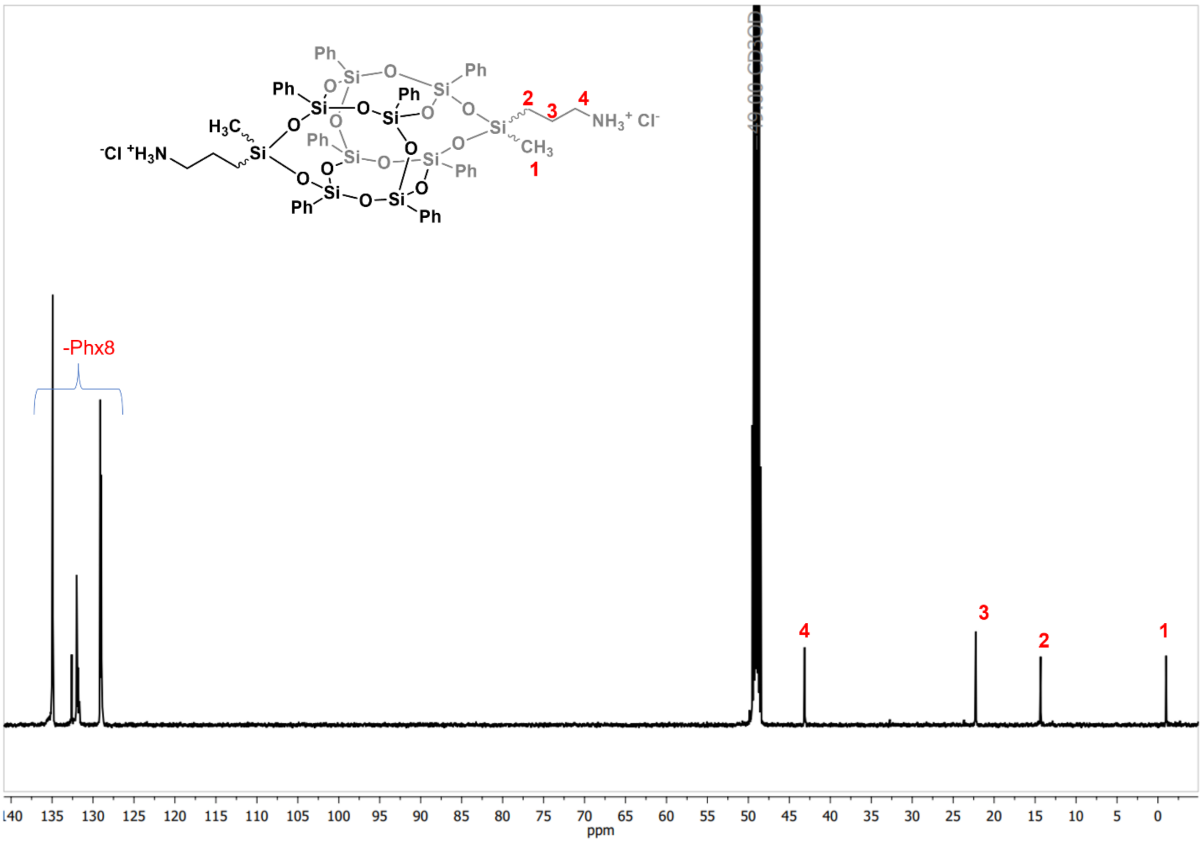

Figure S9. ^{13}C NMR (126 MHz, MeOD, 300 K) spectrum of **1**.

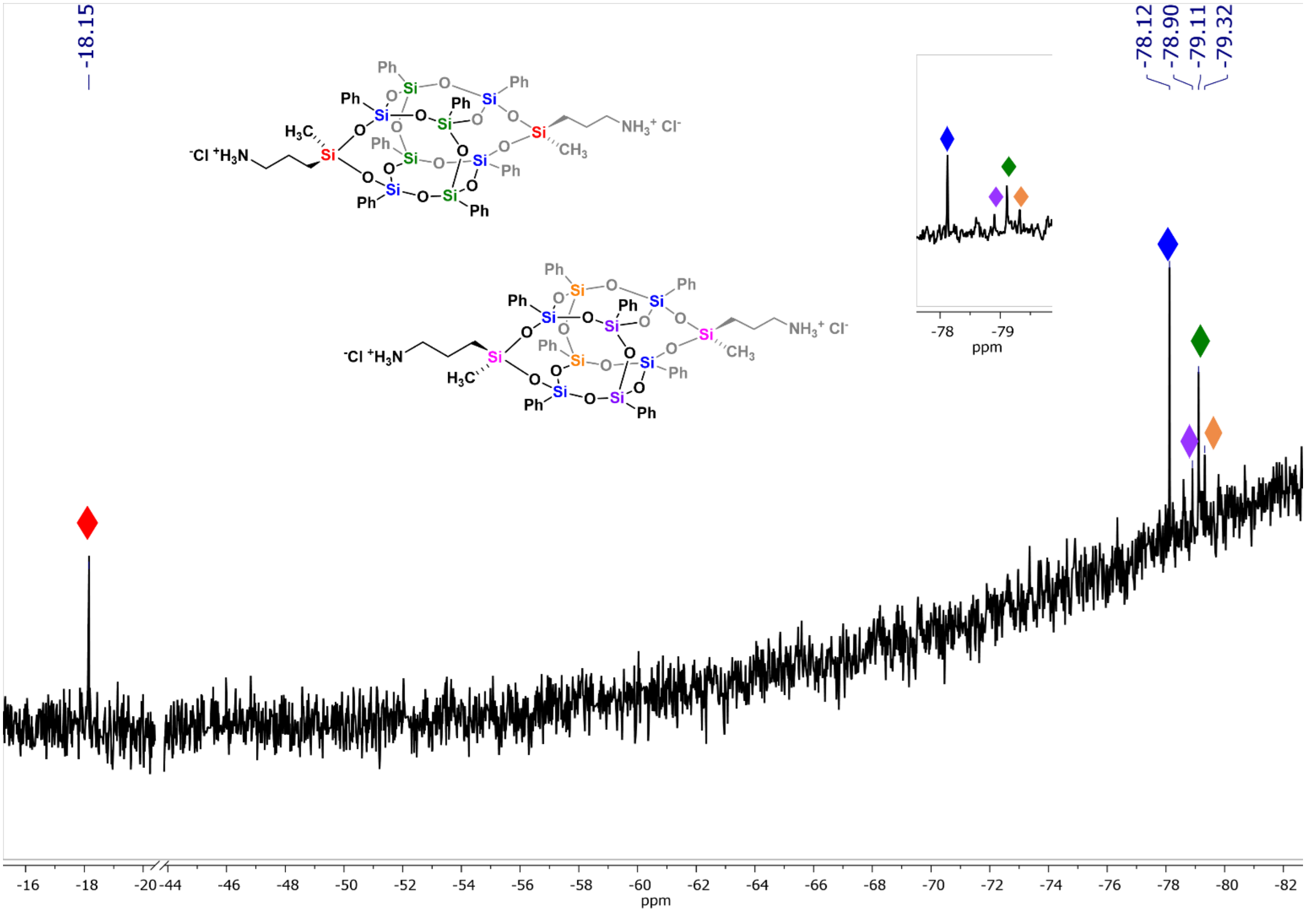

Figure S10. ^{29}Si NMR (99 MHz, MeOD) spectrum of *cis*-**2** and *trans*-**2**.

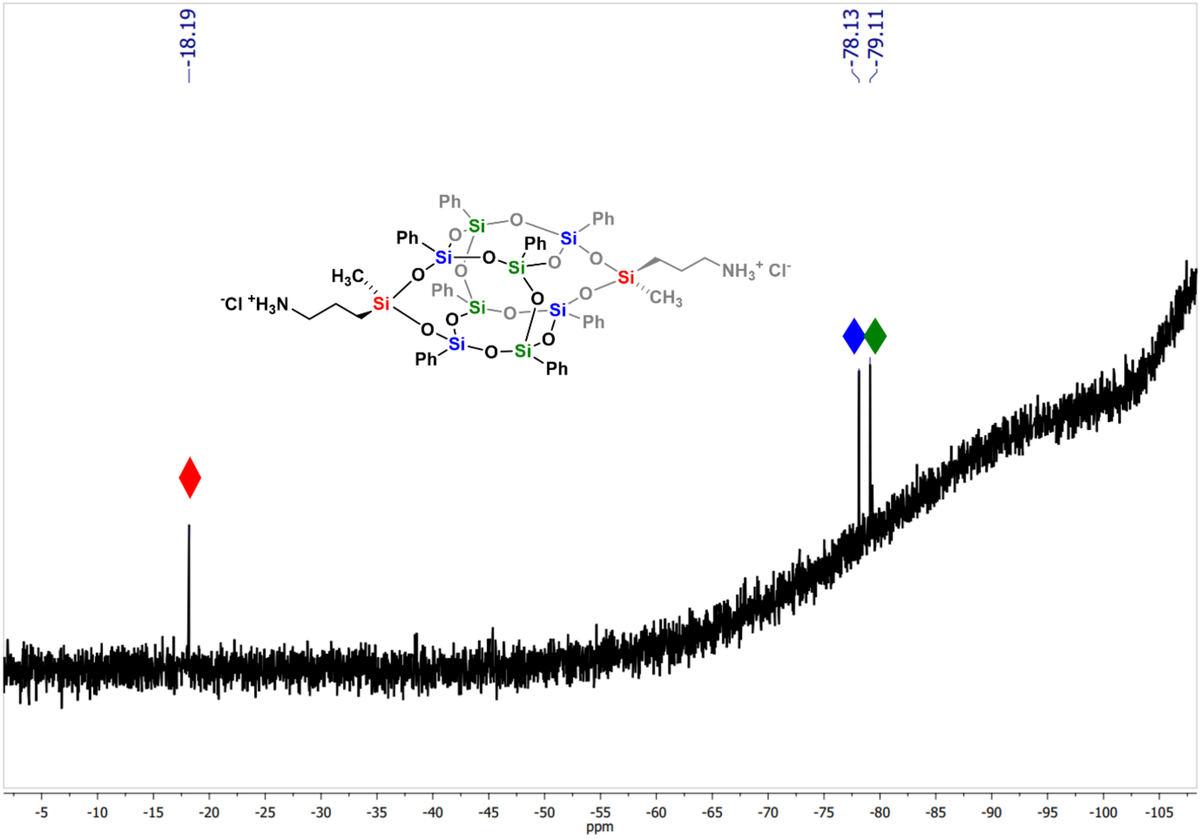

Figure S11. ^{29}Si NMR (99 MHz, MeOD) spectrum of *trans*-**2**.

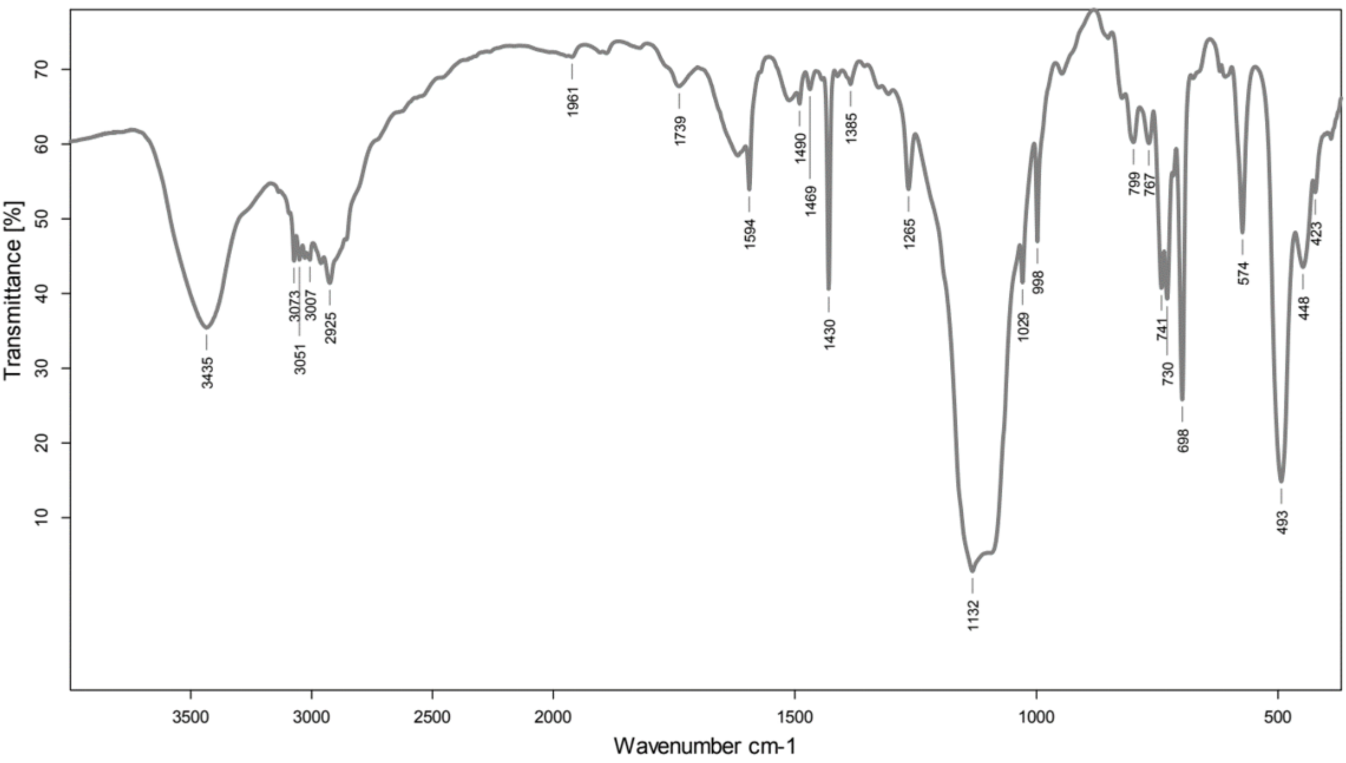

Figure S12. FT-IR (KBr pellet) spectrum of **2**.

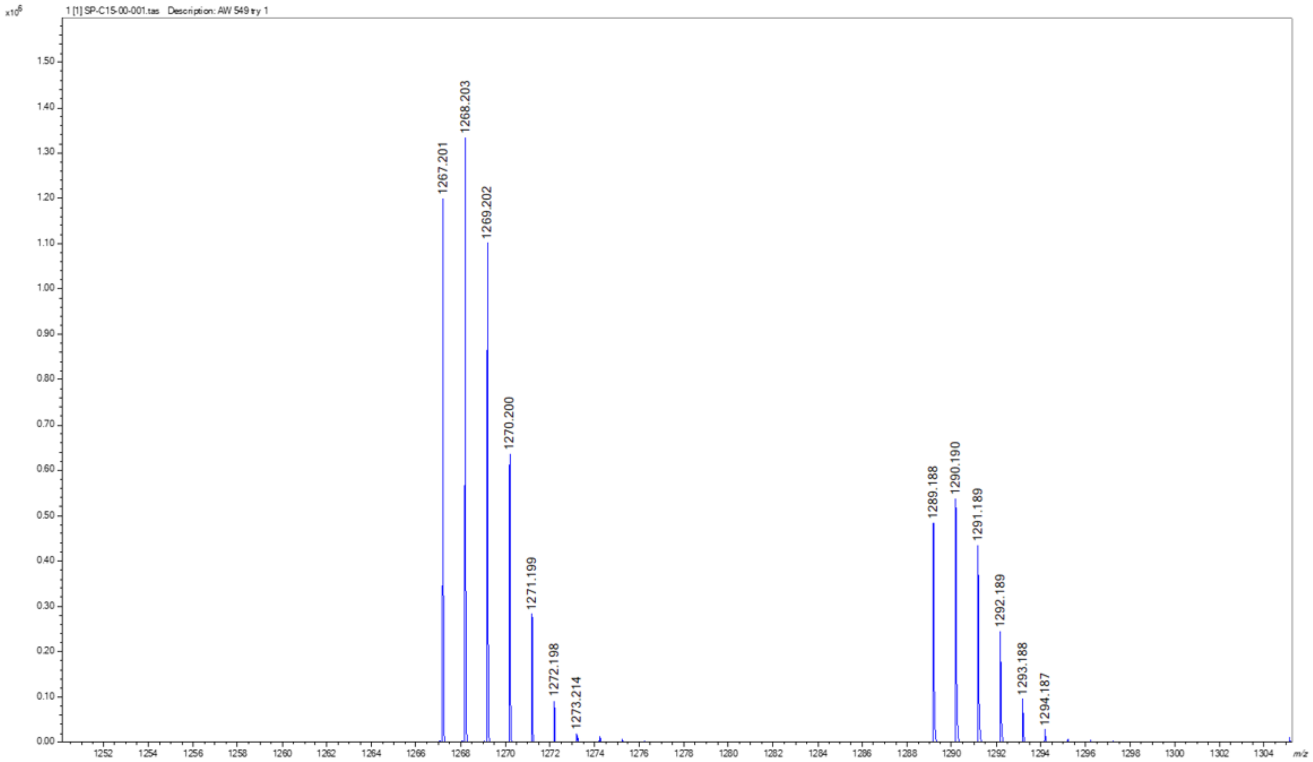

Figure S13. MALDI-TOF MS spectrum of **2**.

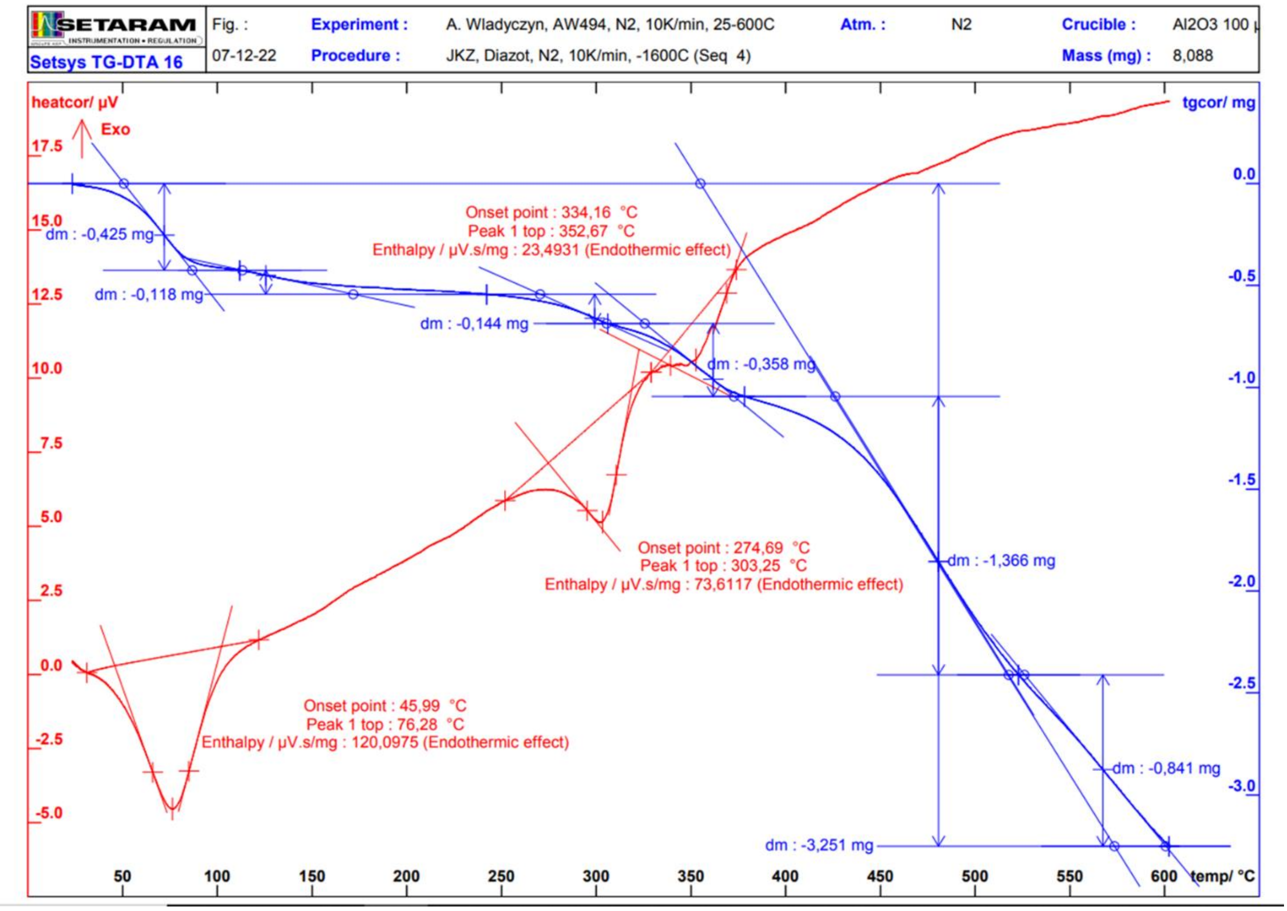

Figure S14. TGA-DTA diagram of **2**. Heated 10 K/min under N_2 flow.

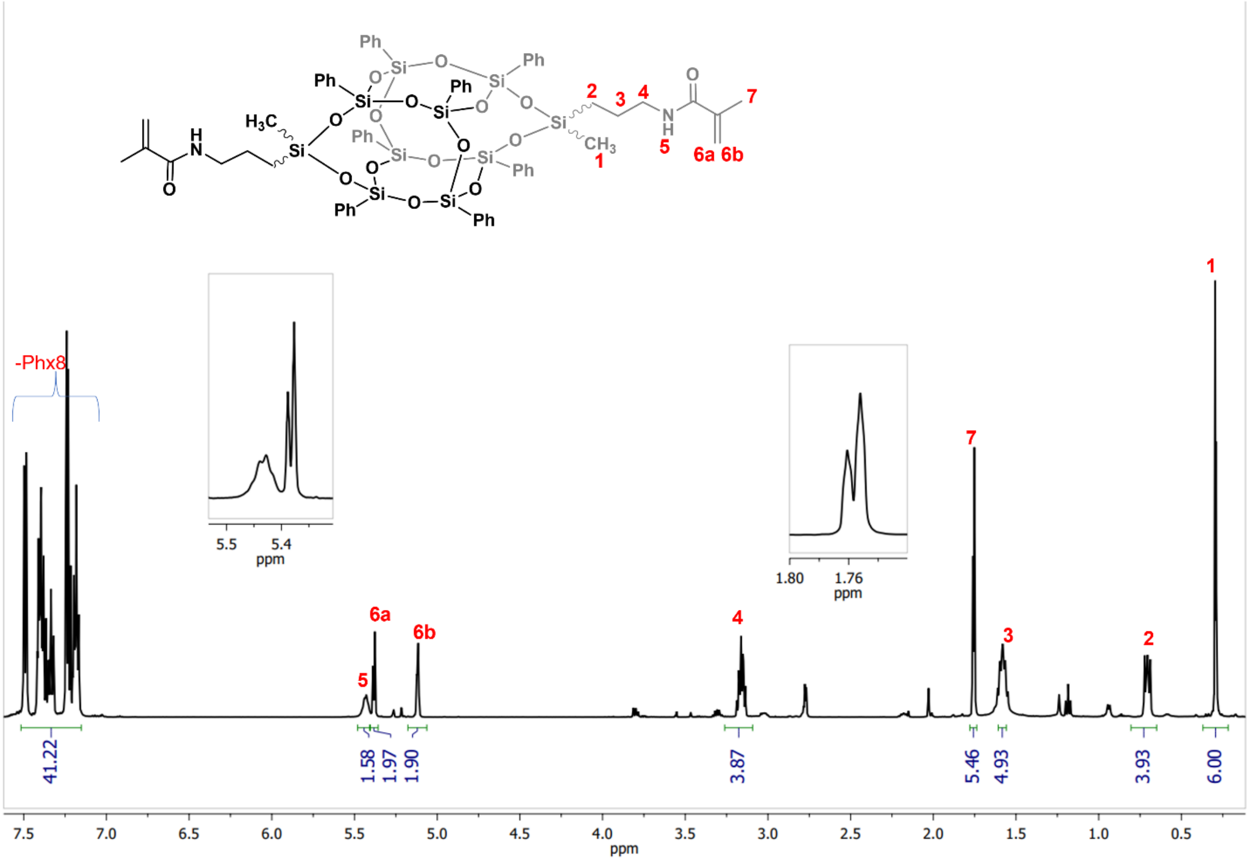

Figure S15. ^{1}H NMR (500 MHz, $CDCl_3$, 300 K) spectrum of **3**.

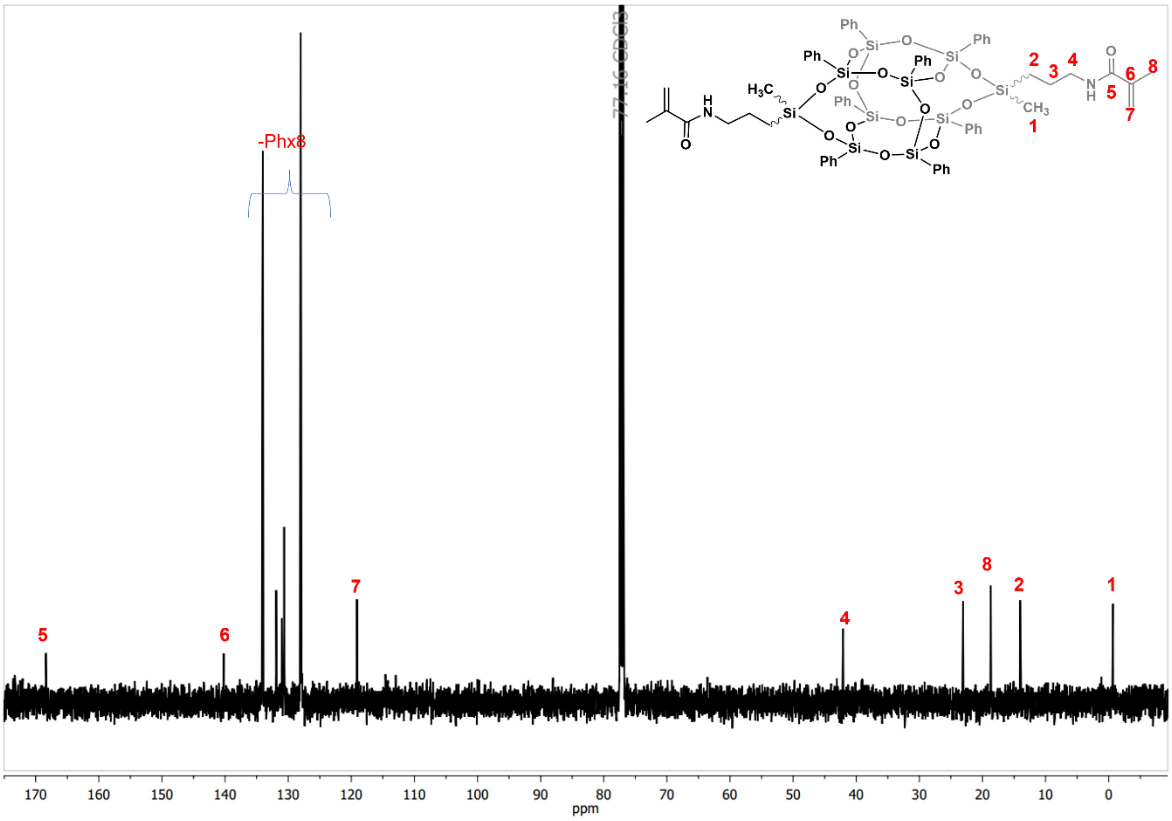

Figure S16. ^{13}C NMR (126 MHz, $CDCl_3$, 300 K) spectrum of **3**.

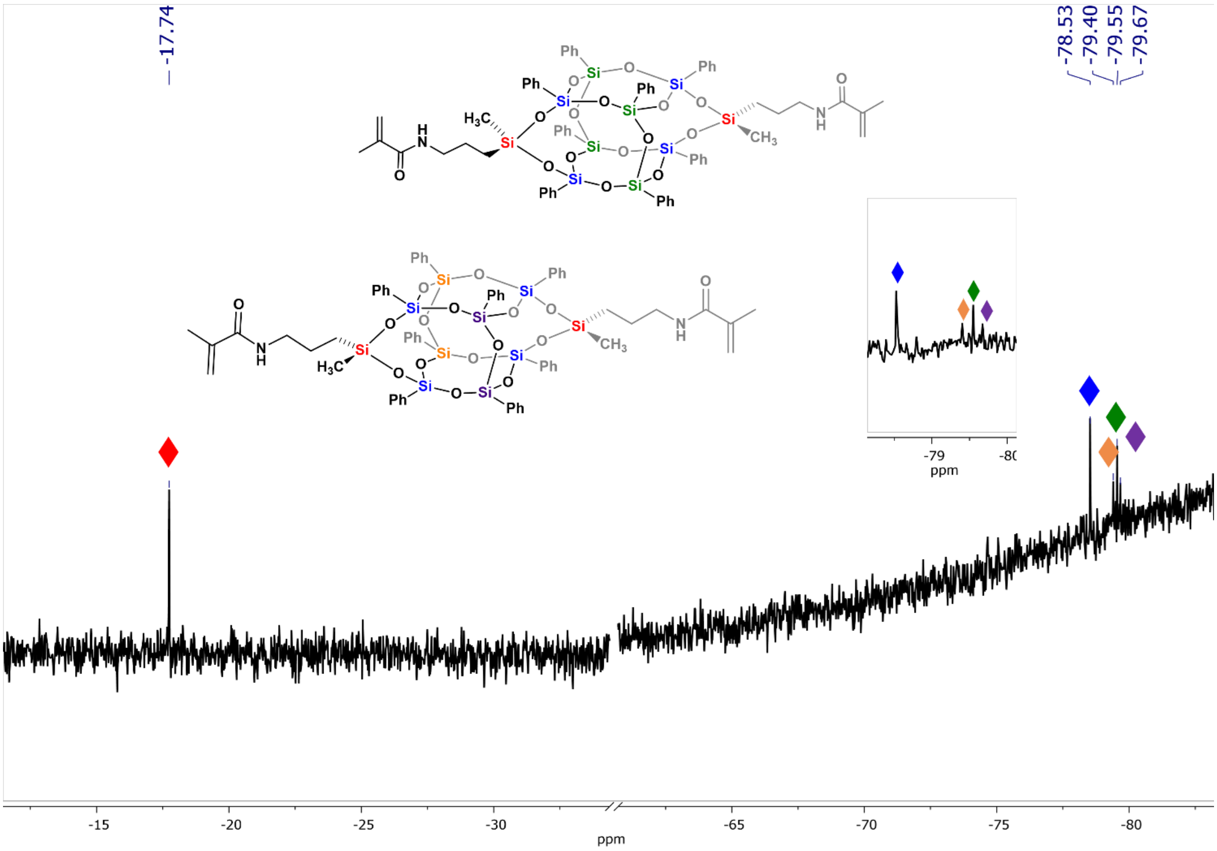

Figure S17. ^{29}Si NMR (99 MHz, $CDCl_3$) spectrum of *cis*-**3** and *trans*-**3**.

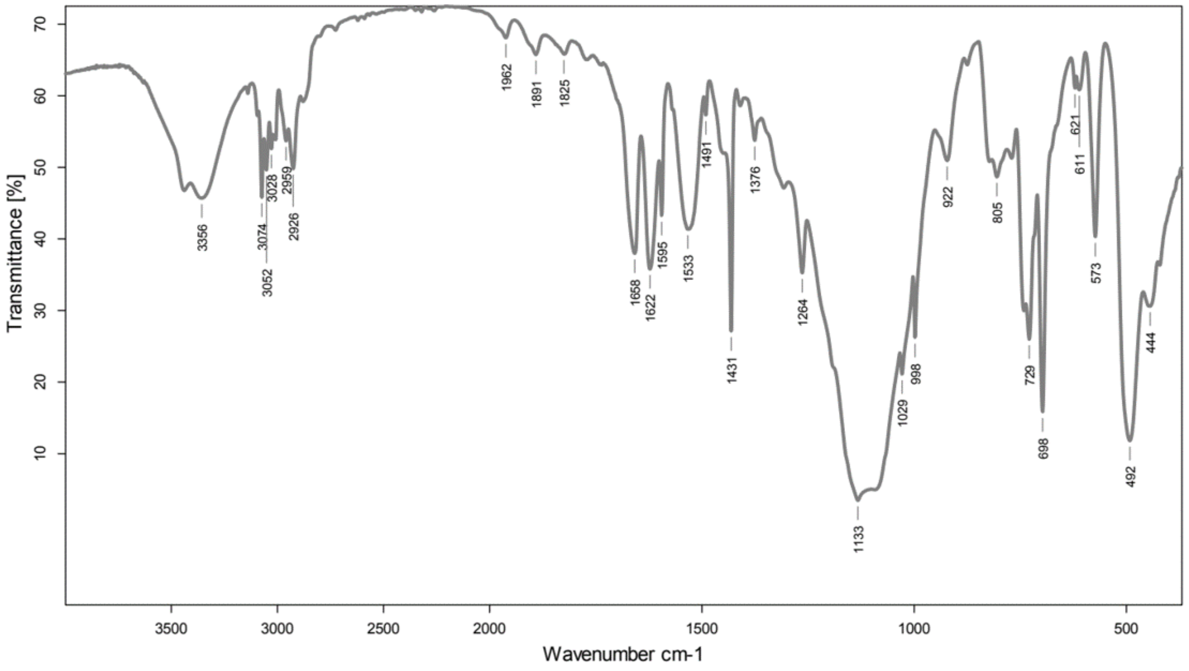

Figure S18. FT-IR (KBr pellet) spectrum of **3**.

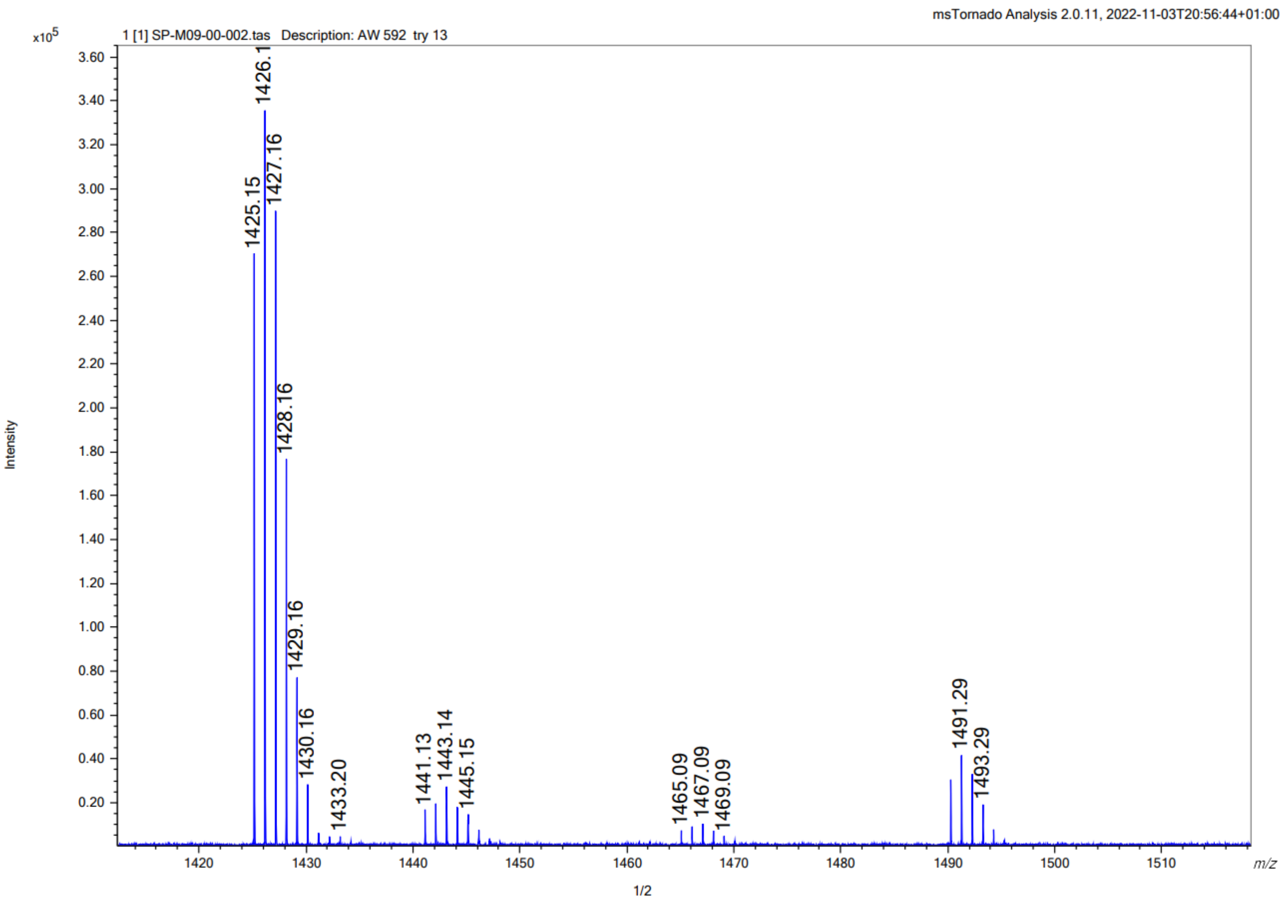

Figure S19. MALDI-TOF MS spectrum of **3**.

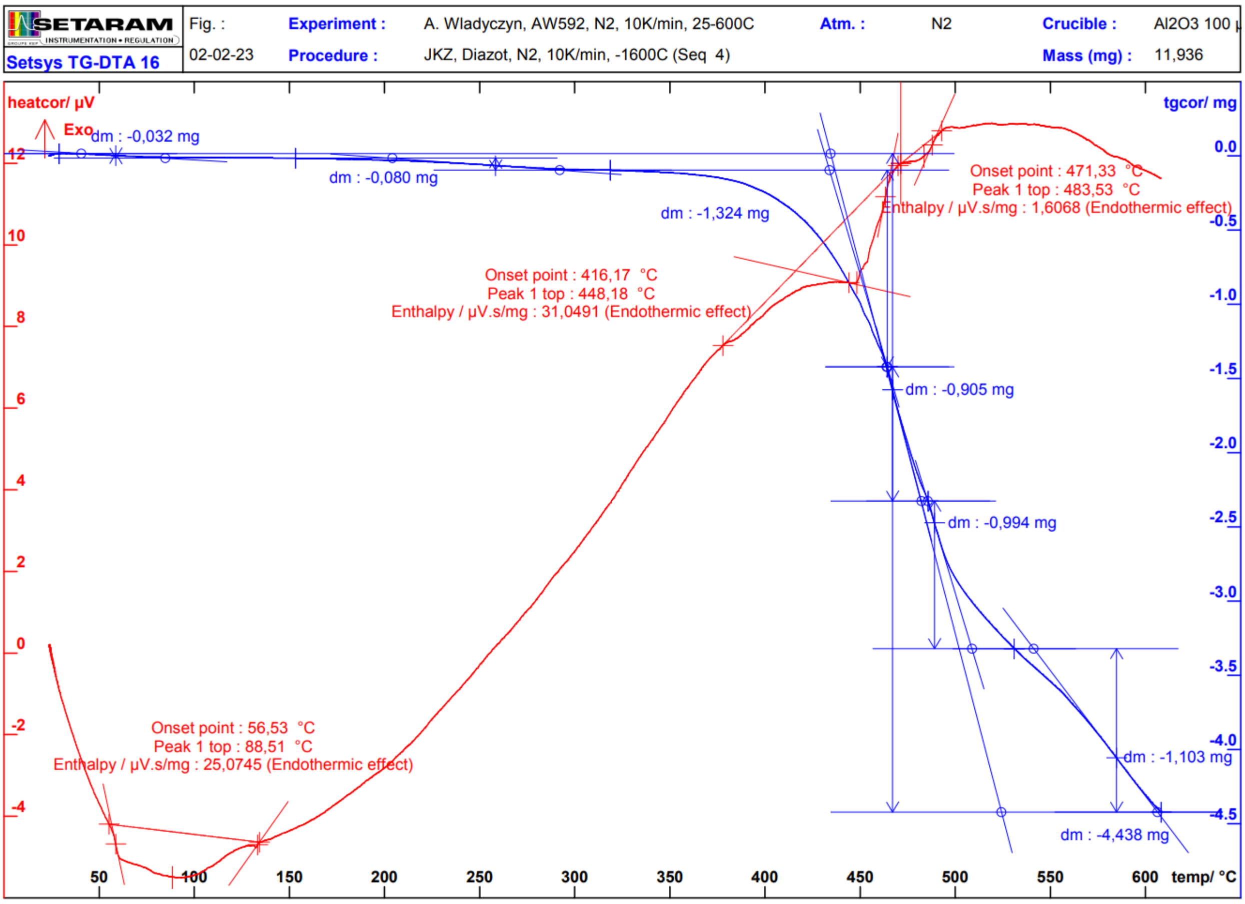

Figure S20. TG-DTA diagram of **3**. Heated 10 K/min under N_2 flow.

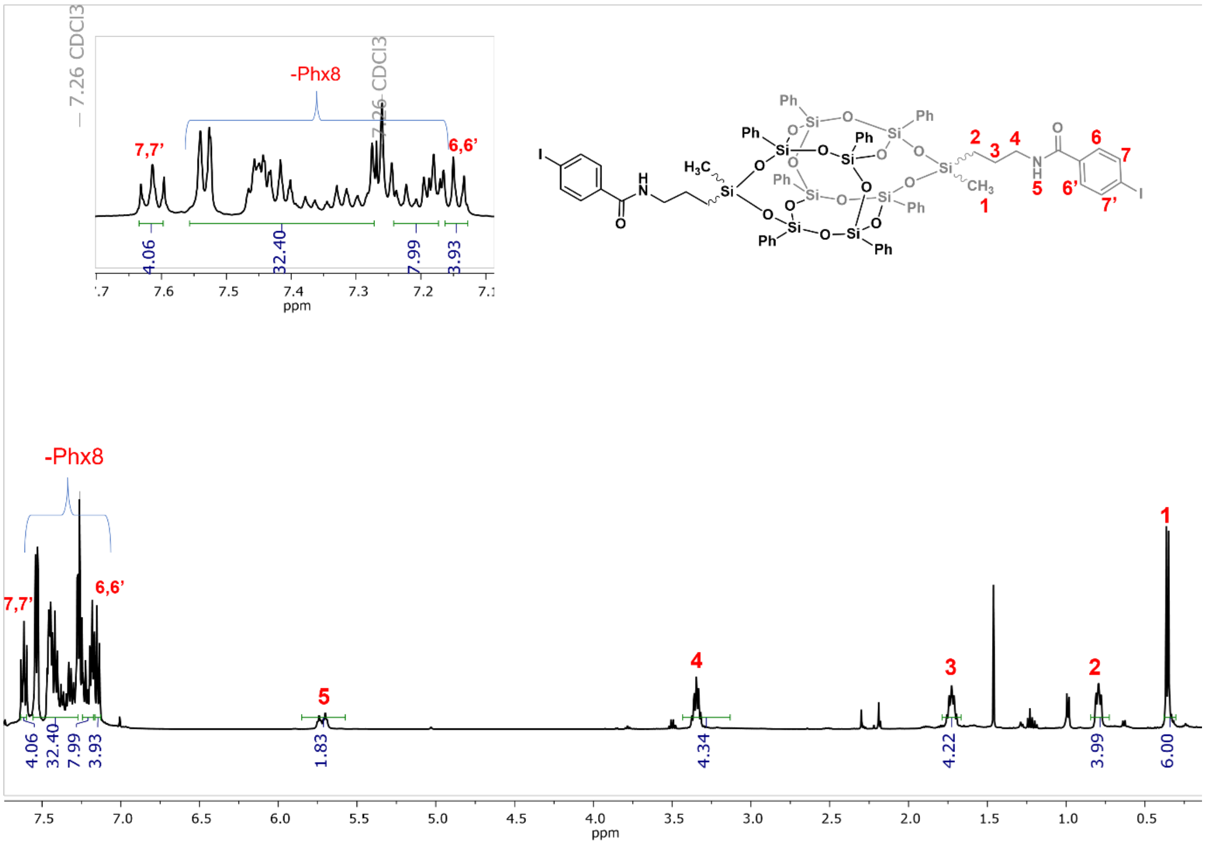

Figure S21. ^{1}H NMR (500 MHz, $CDCl_3$, 300 K) spectrum of **4**.

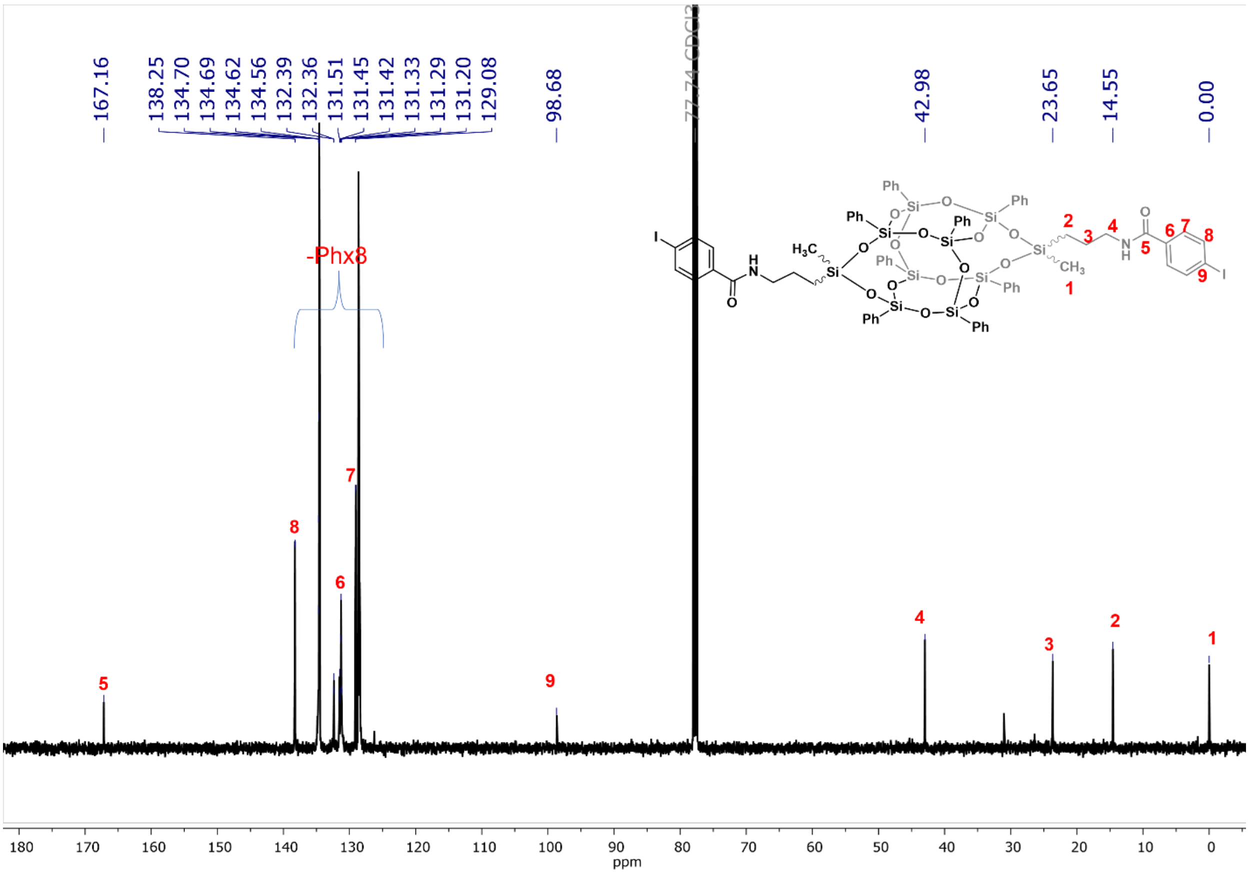

Figure S22. ^{13}C NMR (126 MHz, $CDCl_3$, 300 K) spectrum of **4**.

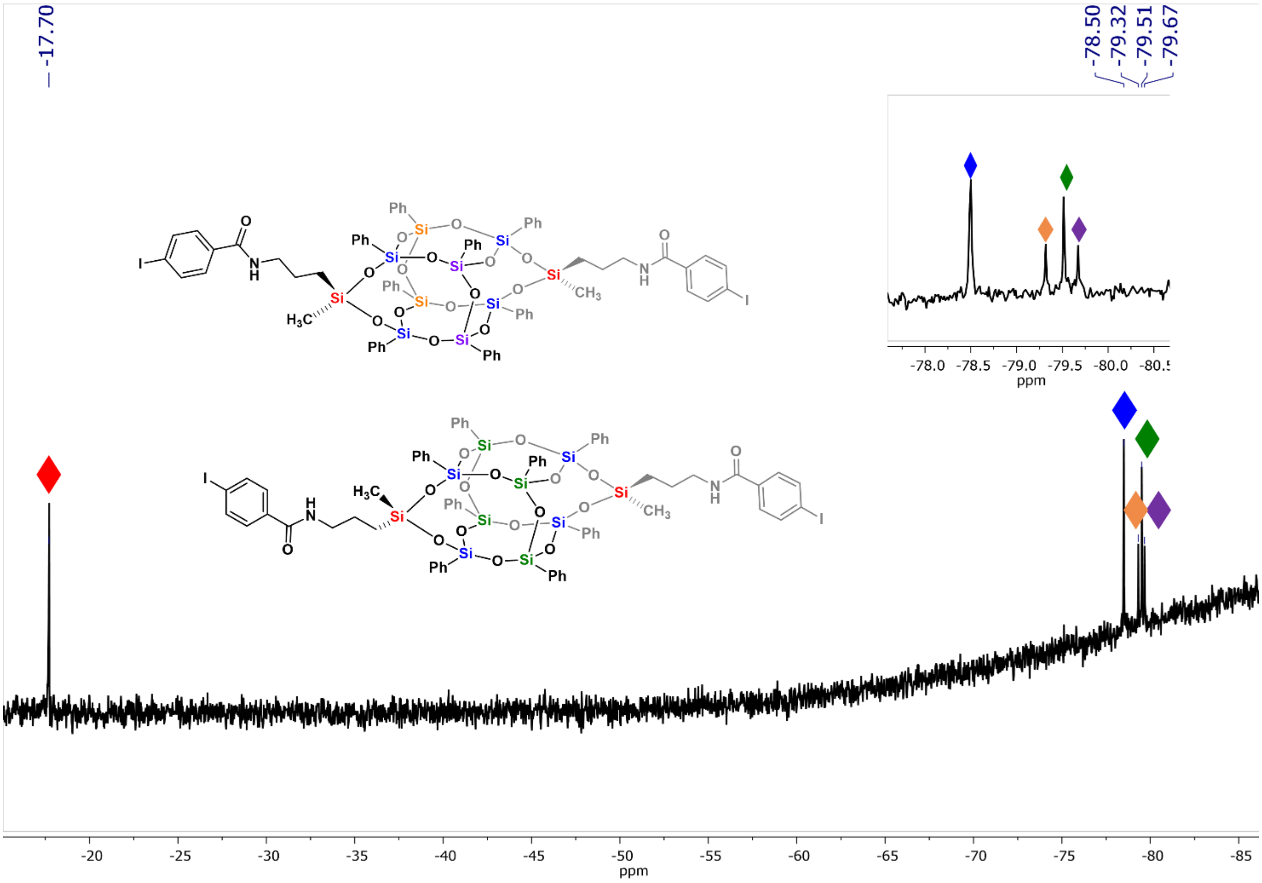

Figure S23. ^{29}Si NMR (99 MHz, $CDCl_3$, 300K) spectrum of **4**.

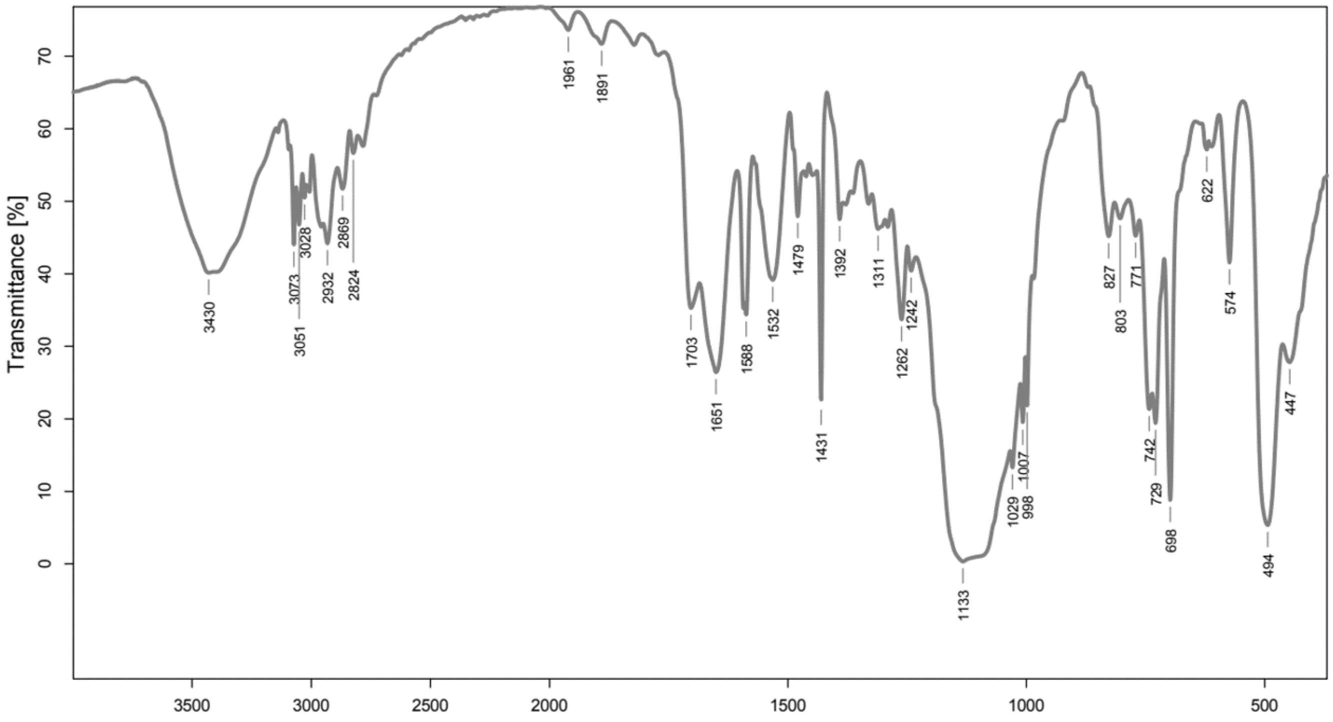

Figure S24. FT-IR (KBr pellet) spectrum of **4**.

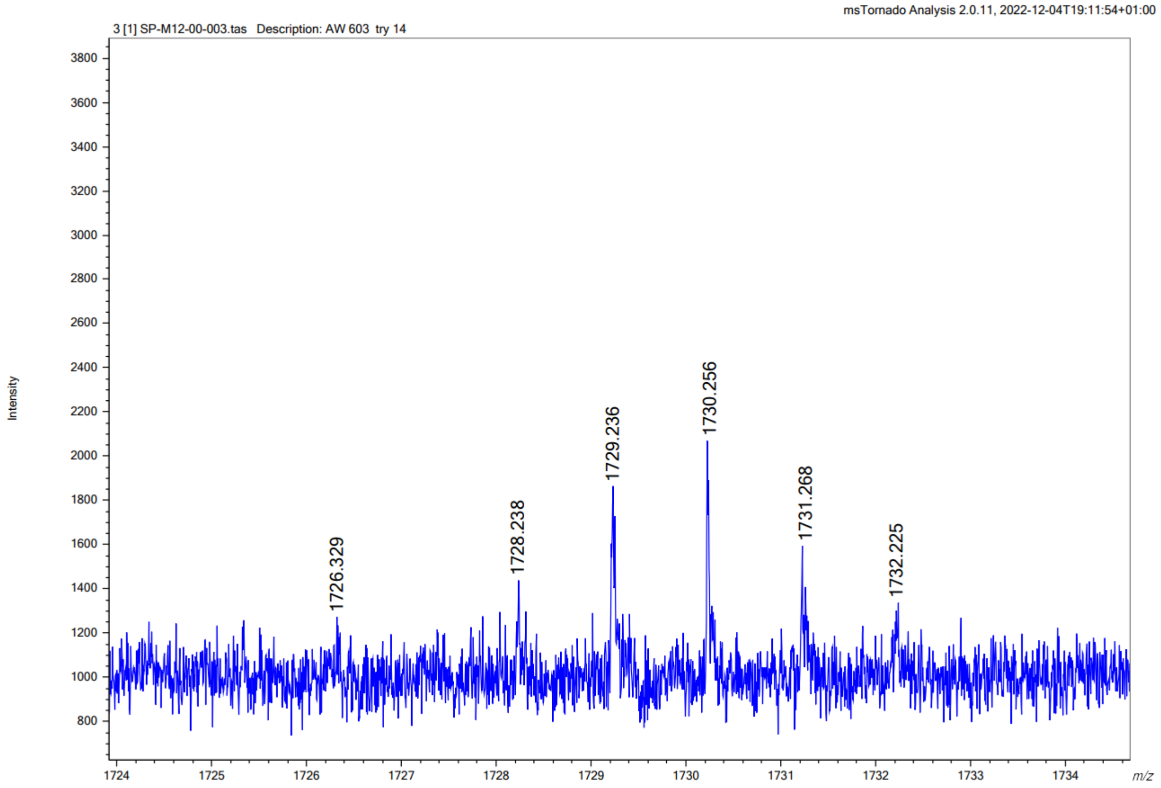

Figure S25. MALDI-TOF MS spectrum of **4**.

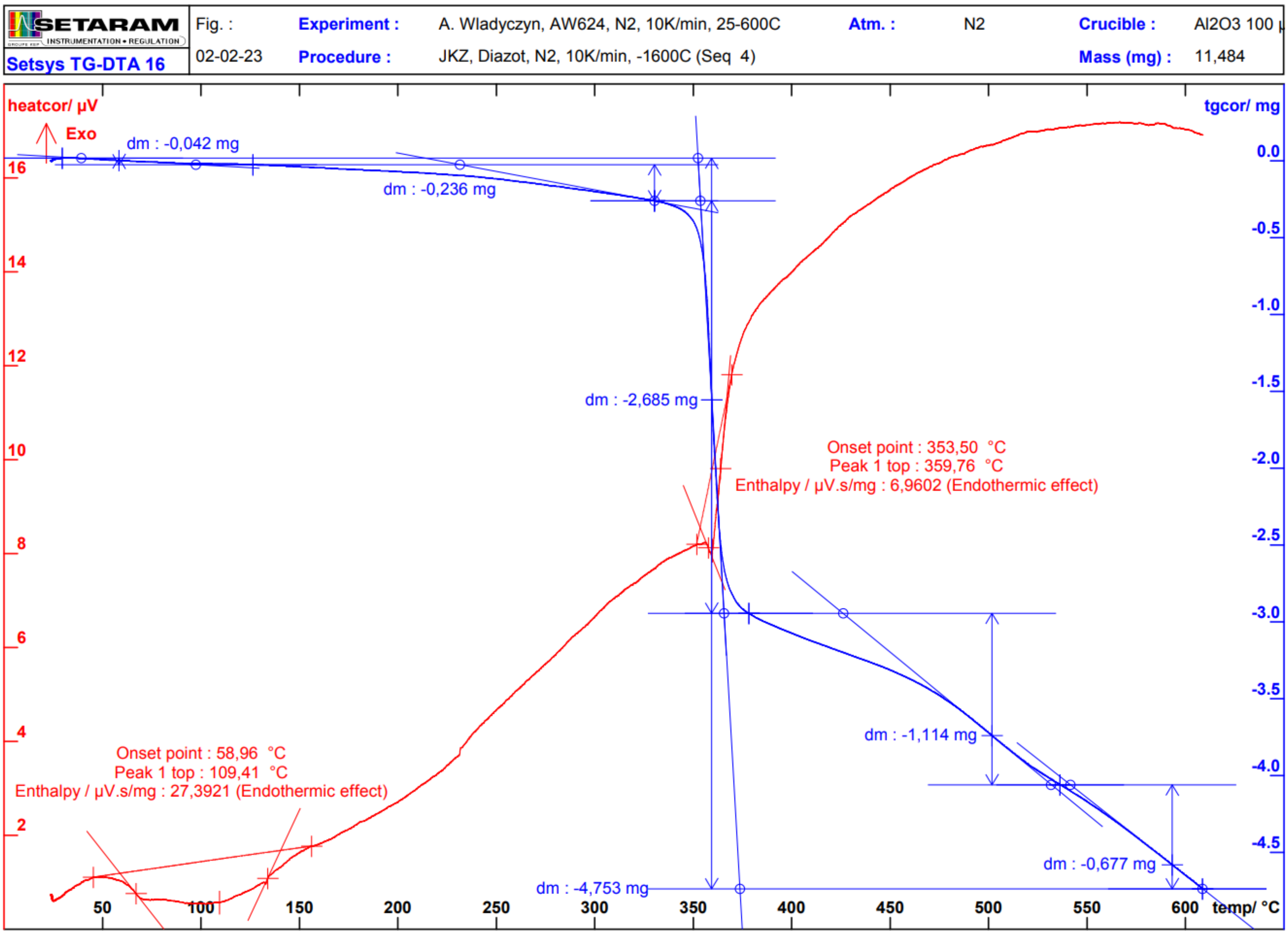

Figure S26. TGA-DTA diagram of **4**. Heated 10 K/min under N_2 flow.

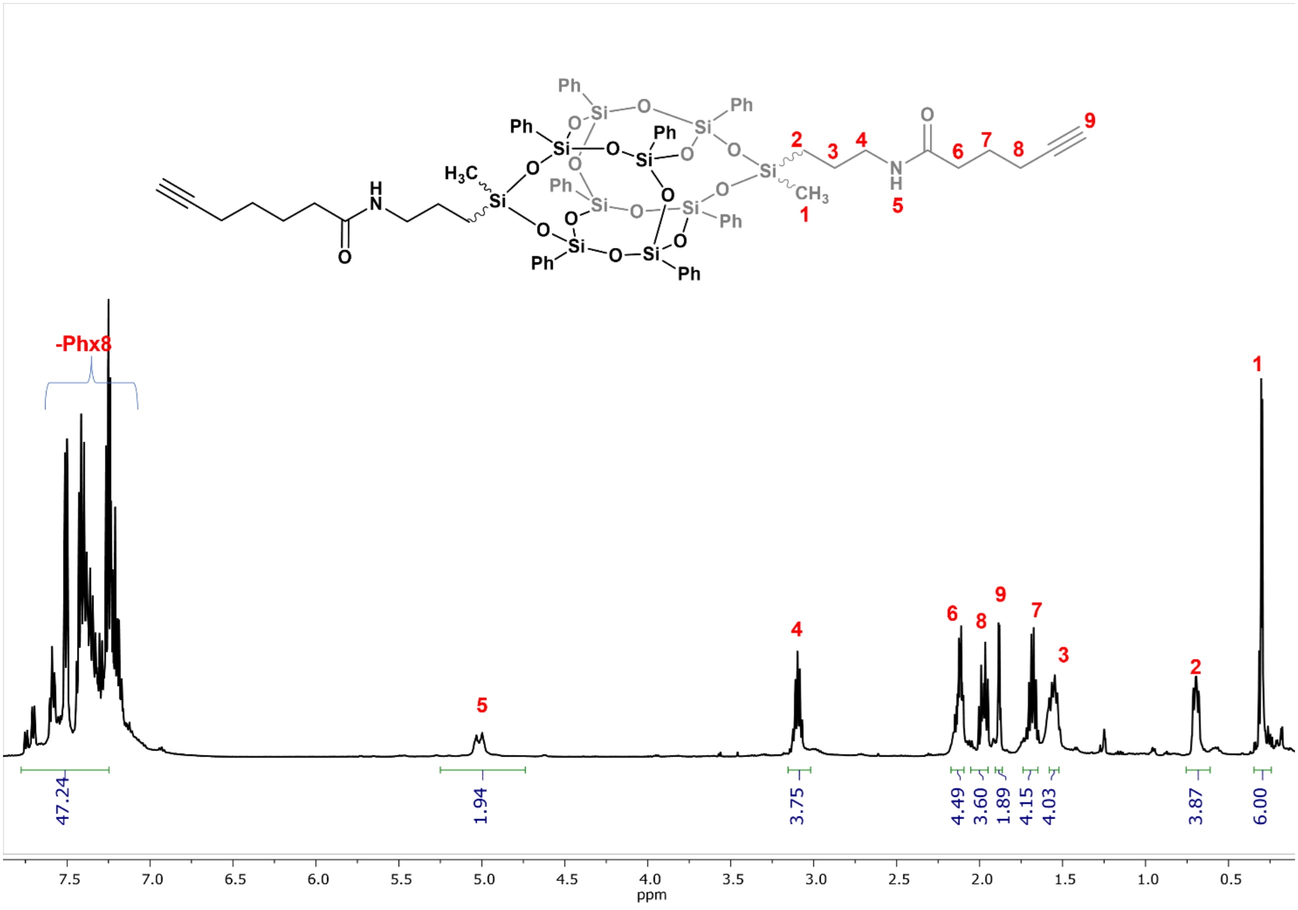

Figure S27. ^{1}H NMR (500 MHz, $CDCl_3$, 300 K) spectrum of **5**.

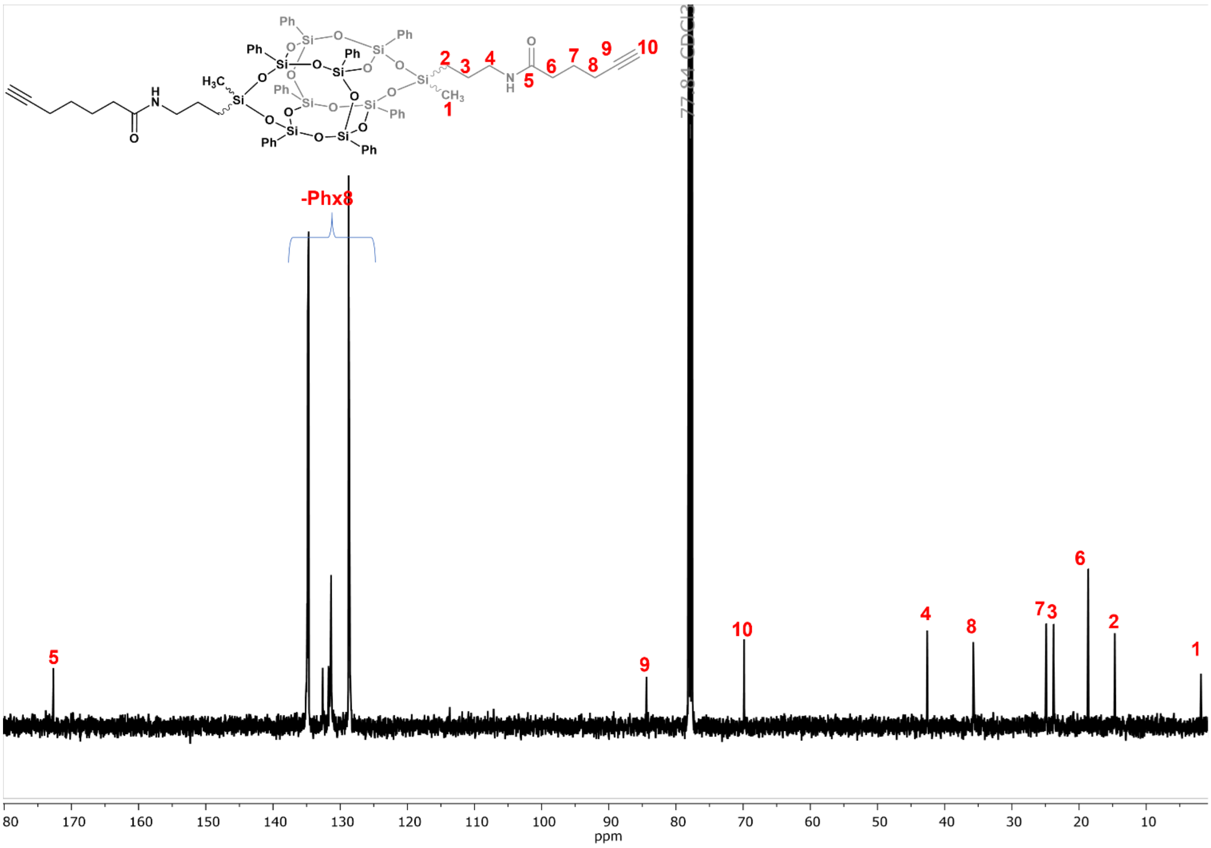

Figure S28. ^{13}C NMR (126 MHz, $CDCl_3$, 300K) spectrum of **5**.

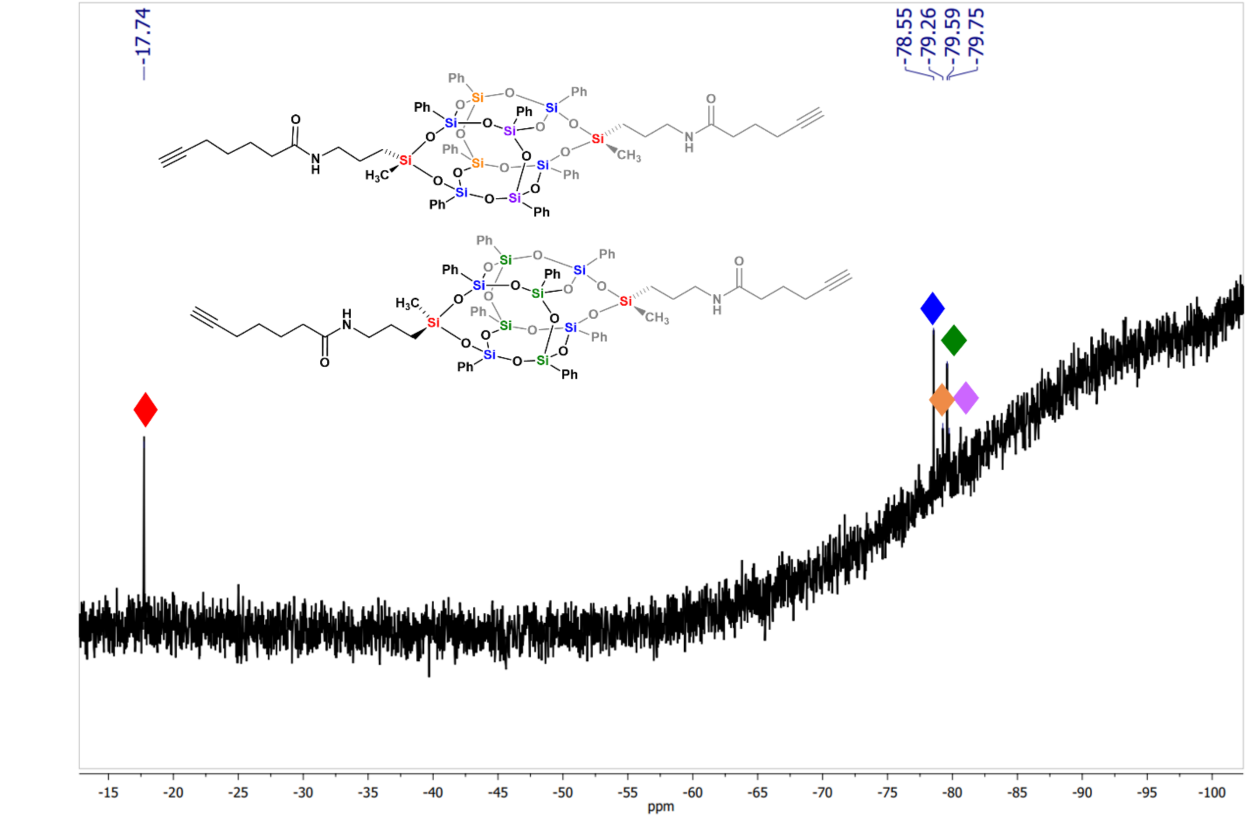

Figure S29. ^{29}Si NMR (99 MHz, $CDCl_3$) spectrum of **5**.

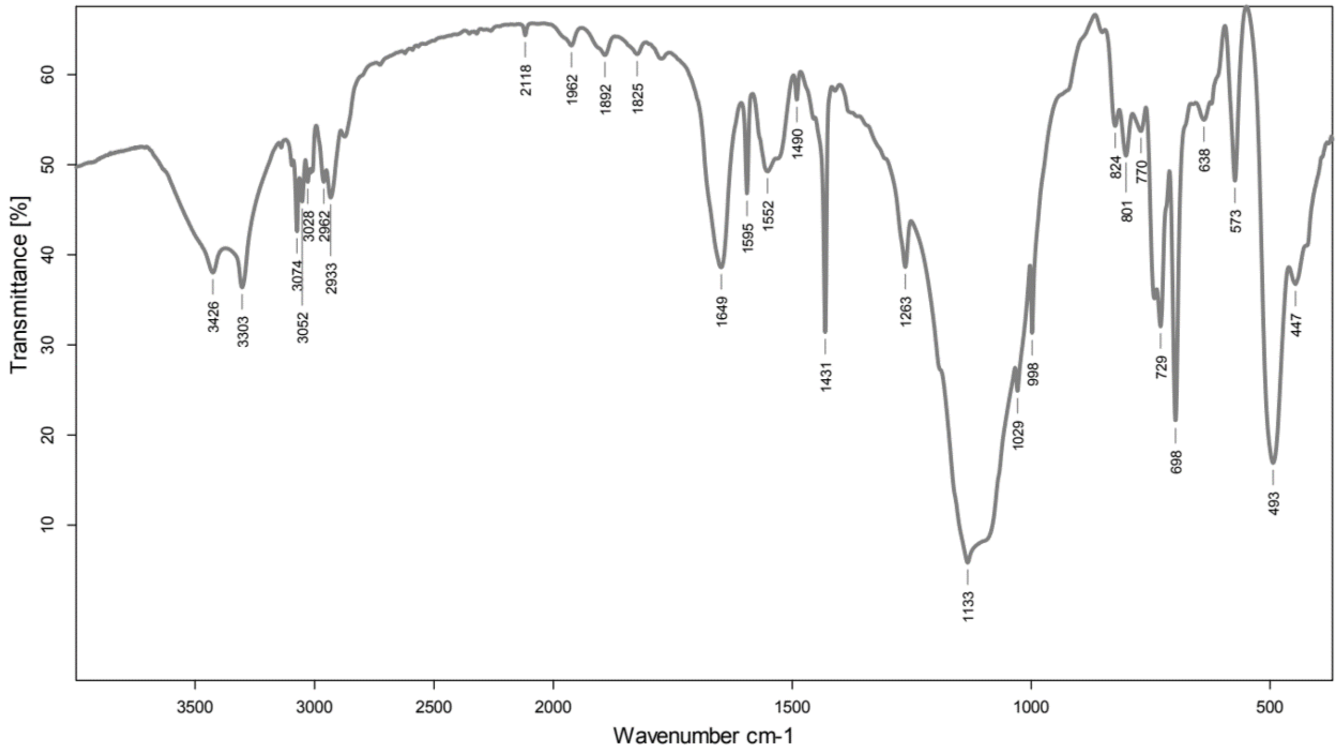

Figure S30. FT-IR (KBr pellet) spectrum of **5**.

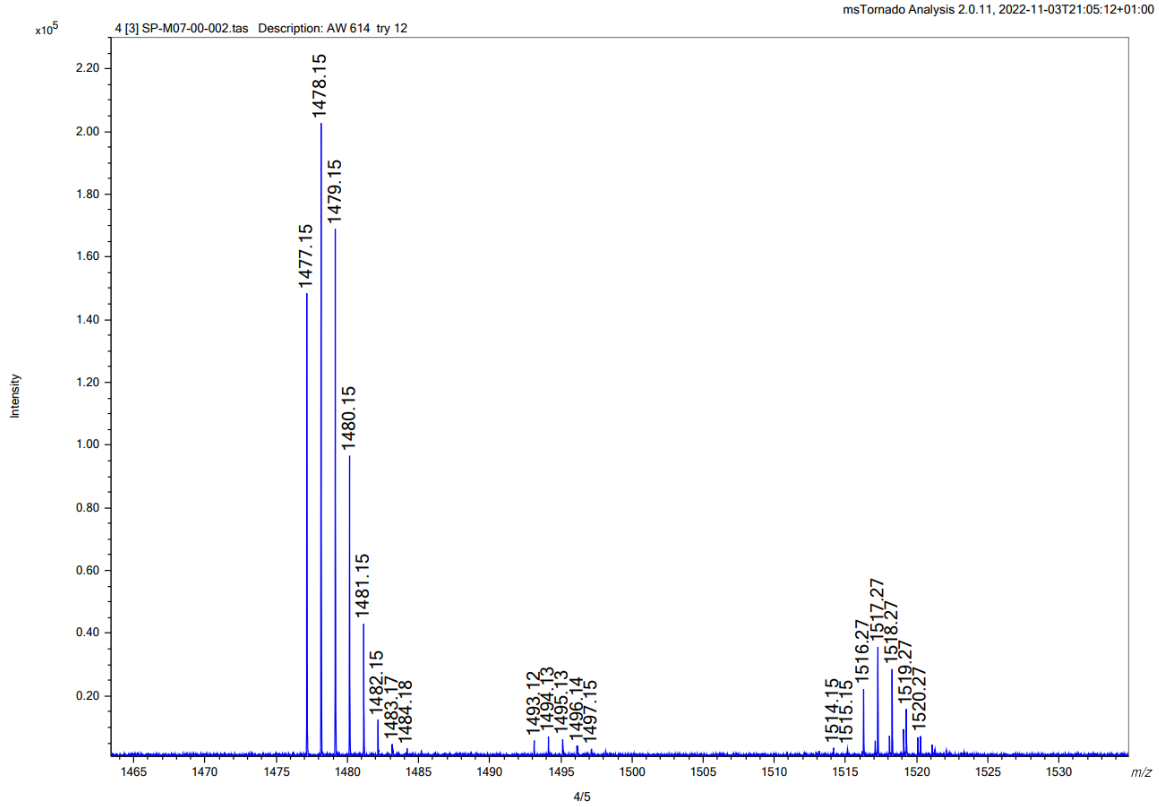

Figure S31. MALDI-TOF MS spectrum of **5**.

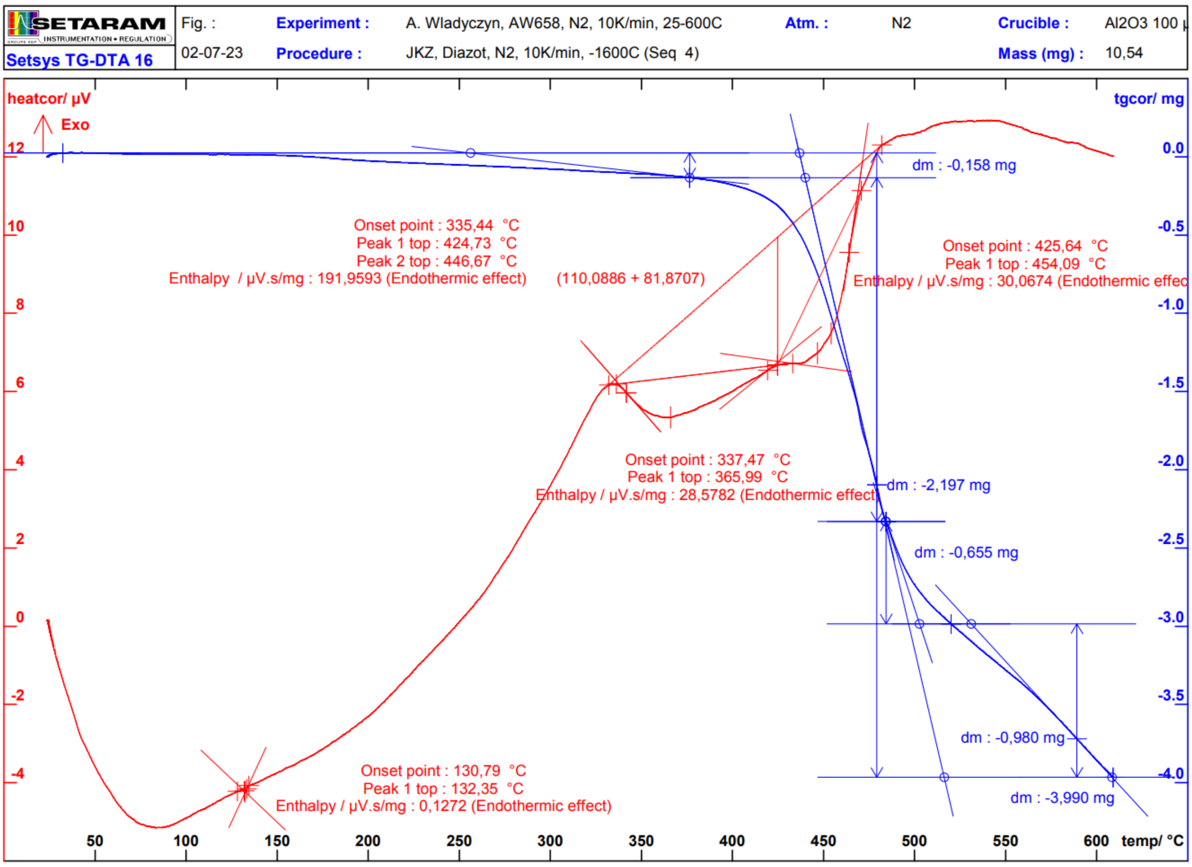

Figure S32. TGA-DTA diagram of **5**. Heated 10 K/min under N_2 flow.

Table S1. Crystal data and structure refinement parameters for **1**, **2** and **3**.[1-5]

No.	1	2	3
CCDC	2211995	2209518	2217825
Chemical formula	$C_{66}H_{78}N_2O_{18}Si_{10}$	$C_{70.11}Cl_{0.5}H_{99.11}N_2O_{20.11}Si_{10}$	$C_{64}H_{70}N_2O_{16}Si_{10}\cdot 2(C_4H_8O)$
Mr	1468.20	1594.73	1548.32
Temperature/K	100.00(10)	100.00(10)	100.00(10)
Crystal system	monoclinic	trigonal	triclinic
Space group	$P2_1/c$	$R\bar{3}$	$P\bar{1}$
a/Å	11.027(4)	40.875(10)	9.969(8)
b/Å	16.624(7)	40.875(10)	13.049(2)
c/Å	19.817(3)	13.253	15.929(7)
α/°	90	90	102.52(6)°
β/°	95.910(10)	90	91.89(2)°
γ/°	90	120	112.30(7)
Volume/Å^3	3613(2)	19176.1(9)	1856(2)
Z	2	9	1
ρ_{calc}g/cm^3	1.349	1.243	1.385
μ/mm^{-1}	2.297	2.142	2.270
F(000)	1544.0	7610.0	816.0
Crystal size/mm^3	0.128 × 0.069 × 0.054	0.253 × 0.153 × 0.101	0.206 × 0.189 × 0.169
Radiation	Cu Kα	Cu Kα	Cu Kα
2Θ range for data collection/°	6.956 to 146.786	8.334 to 147.286	7.558 to 147.166
Index ranges	-7 ≤ h ≤ 12, -20 ≤ k ≤ 20, -24 ≤ l ≤ 24	-50 ≤ h ≤ 47, -49 ≤ k ≤ 48, -9 ≤ l ≤ 15	-12 ≤ h ≤ 11, -16 ≤ k ≤ 16, -19 ≤ l ≤ 19
Reflections collected	36442	42261	21199
Independent reflections	6989 [R_{int} = 0.0305, R_{sigma} = 0.0283]	8274 [R_{int} = 0.0499, R_{sigma} = 0.0319]	6935 [R_{int} = 0.0339, R_{sigma} = 0.0321]
Data/restraints/parameters	6989/0/441	8274/196/449	6935/404/643
Goodness-of-fit on F^2	1.026	1.156	1.004
Final R indexes [I>=2σ (I)]	R1 = 0.0349, wR2 = 0.0843	R_1 = 0.1023, wR_2 = 0.2917	R1 = 0.0851, wR2 = 0.2250
Final R indexes [all data]	R1 = 0.0466, wR2 = 0.0891	R_1 = 0.1282, wR_2 = 0.3205	R1 = 0.1055, wR2 = 0.2495
Largest diff. peak/hole / e Å^{-3}	0.44/-0.29	0.57/-0.47	0.69/-0.44

Computer programs: *CrysAlis PRO* 1.171.42.42a (Rigaku OD, 2022), olex2.solve 1.5 (Bourhis *et al.*, 2015), *SHELXL* 2019/2 (Sheldrick, 2015), Olex2 1.5 (Dolomanov *et al.*, 2009).

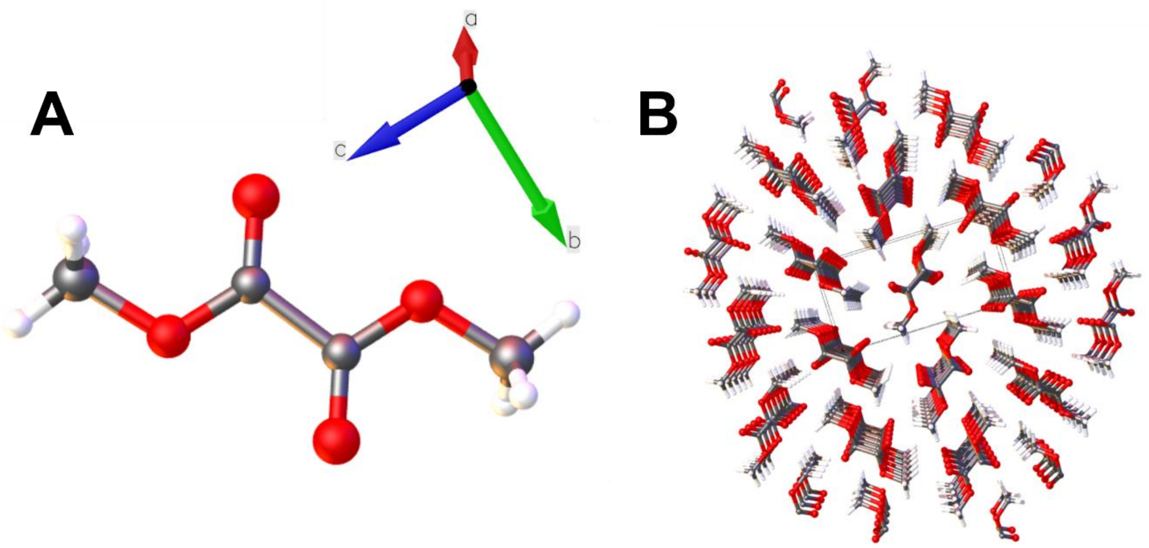

Figure S32. (A) Crystal structure of dimethyl oxalate. (B) Fragment of its crystal packing. Silver, carbon; red, oxygen; white; hydrogen. Thermal ellipsoids are shown at the 50% probability level.

Table S2. Crystal data and structure refinement parameters for dimethyl oxalate.

Empirical formula	$C_8H_{12}O_8$
Formula weight	236.18
Temperature/K	100.01(10)
Crystal system	monoclinic
Space group	$P2_1/n$
a/Å	3.7883(3)
b/Å	11.7436(10)
c/Å	6.1736(5)
α/°	90
β/°	104.776(9)
γ/°	90
Volume/Å^3	265.57(4)
Z	1
ρ_{calc}g/cm^3	1.477
μ/mm^{-1}	0.135
F(000)	124.0
Crystal size/mm^3	0.155 × 0.138 × 0.387
Radiation	Mo Kα (λ = 0.71073)
2Θ range for data collection/°	6.94 to 57.38
Index ranges	$-3 \leq h \leq 4, -15 \leq k \leq 7, -8 \leq l \leq 6$
Reflections collected	1002
Independent reflections	594 [R_{int} = 0.0148, R_{sigma} = 0.0341]
Data/restraints/parameters	594/0/18
Goodness-of-fit on F^2	1.096
Final R indexes [I>=2σ (I)]	R_1 = 0.0769, wR_2 = 0.2063
Final R indexes [all data]	R_1 = 0.0895, wR_2 = 0.2215
Largest diff. peak/hole / e Å^{-3}	0.60/-0.63

References

1. Kratzert, D.; Krossing, I. Recent improvements in DSR. *J. Appl. Cryst.* **2018**, *51*, 928–934.
2. Dolomanov, O. V.; Bourhis, L. J.; Gildea, R. J.; Howard, J. A. K.; Puschmann, H. OLEX2: A complete structure solution, refinement and analysis program. *J. Appl. Cryst.* **2009**, *42*, 339–341.
3. Kratzert, D.; Holstein, J. J.; Krossing, I. DSR: enhanced modelling and refinement of disordered structures with SHELXL. *J. Appl. Cryst.* **2015**, *48*, 933–938.
4. Sheldrick, G. M. A short history of SHELX. *Acta Cryst.* **2008**, *A64*, 112–122.
5. Sheldrick, G. M. Crystal Structure Refinement with SHELXL. *Acta Cryst.* **2015**, *C71*, 3–8.
